# Penta band single negative meta-atom absorber designed on square enclosed star-shaped modified split ring resonator for S-, C-, X- and Ku- bands microwave applications

**DOI:** 10.1038/s41598-021-87958-6

**Published:** 2021-04-22

**Authors:** Md. Rashedul Islam, Mohammad Tariqul Islam, Md. Moniruzzaman, Md. Samsuzzaman, Haslina Arshad

**Affiliations:** 1 Department of Electrical, Electronic and Systems Engineering, Faculty of Engineering and Built Environment, Universiti Kebangsaan Malaysia (UKM), 43600 Bangi, Selangor Malaysia; 2 Department of Computer and Communication Engineering, Faculty of Computer Science and Engineering, Patuakhali Science and Technology University, Patuakhali, Bangladesh; 3 Institute of IR4.0, Universiti Kebangsaan Malaysia (UKM), 43600 Bangi, Selangor Malaysia

**Keywords:** Materials science, Physics

## Abstract

This paper represents a penta band square enclosed star-shaped modified split ring resonator (SRR) based single negative meta-atom absorber (MAA) for multi-band microwave regime applications. FR-4 low-cost material has been used as a substrate to make the MAA unit cell with 0.101λ_0_ × 0.101λ_0_ of electrical size, where λ_0_ is the wavelength calculated at the lower resonance frequency of 3.80 GHz. There are two outer square split ring and one inner star ring shape resonator of 0.035 mm thickness of copper placed on the one side, and another side of the substrate has full copper to construct the desired unit cell. The MAA unit cell provides five absorption peaks of 97.87%, 93.65%, 92.66%, 99.95%, and 99.86% at the frequencies of 3.80, 5.65, 8.45, 10.82, and 15.92 GHz, respectively, which covers S-, C-, X-, and Ku- bands. The properties of MAA have been investigated and analyzed in the E-, H-fields and surface current. The EMR and highest Q factor of the designed MAA is 9.87 and 30.41, respectively, and it shows a single negative (SNG) property. Different types of parametric analysis have been done to show the better performance of absorption. Advanced Designed System (ADS) software has been used for equivalent circuit to verify the simulated S_11_ result obtained from the CST-2019 software. Experimental outcomes of the MAA unit cell have a good deal with the simulated result and measured result of the 24 × 20 array of unit cells also shown. Since the unit cell provides superior EMR, excellent Q-factor, and highest absorption so the recommended MAA can be effectively used as a penta band absorber in microwave applications, like notch filtering, sensing, reducing the unintended noise generated with the copper component of the satellite and radar antennas.

## Introduction

The meta-atom is a synthetic arrangement along with excellent substantial properties. The meta-atom is a superb discovery in the modern era, which has been attracted the researchers in the whole world. Most of the work in the electrical engineering, material science, physics and optics communities has underscored the development of effective meta-atoms. Meta-atom is the unit cell of the metamaterial (MTM). The meta-atom structure is mostly constituted of sub-wavelength array components that can adapt the propagation characteristics of electromagnetic (EM) waves. Unique EM properties such as negative refractive index, EM stealth, ideal prism and excellent absorption have been achieved through the use of meta-atoms. These applications involve the field of filters^1–4^, antennas, multiband components^5^, components for bandwidth enhancement^6^, absorber design^7^, leaky wave antennas^8^, SAR reduction^9^, super lenses etc.

Meta-atom absorbers (MAAs) are modern innovations in the field of EM wave appliances such as 5G antenna, reduction cross-section of radar, remote sensing, and photoelectron absorption. MAAs are sort of materials that typically show either negative permeability or negative permittivity, or both are negative as EM waves pass through them. Therefore, they absorb maximum EM waves since the transmission of the MAA is marginal, and the coefficient of reflection is low. Perfect metamaterial absorber (PMA) with split-ring resonator was primarily initiated by Landy et al. in 2008^10^. The absorbing band of this absorber is X, and the peak of absorption is 88% at the 11.5 GHz frequency. After that, intensive attention is paid to researchers, and several metamaterial absorbers (MMAs) have been suggested. An MTM-based absorber is designed to be used for the purpose of pressure, temperature, density, and, humidity sensing in^11^. Two absorption peaks are observed at 6.46, and 7.68 GHz and the absorption rate are more than 90%. Hoque et al. introduced a meta absorber for the applications of X- and Ku-bands with absorption 82.31% at the frequency range 11.56–11.64 GHz, 98.91% at the frequency range 12.15–12.37 GHz and 97.79% at the range of frequency 14–14.3 GHz^12^. The dimension of this absorber unit cell is 15 × 12 mm^2^. A V-shaped metamaterial absorber by an absorption peak of 98.38% at 15.52 GHz is presented in^13^. This metamaterial is built on an FR-4 substrate with a dimension of 8 × 8 mm^2^. Closed square ring resonator-based metamaterial absorber presented in^14^. Owing to its diagonal symmetrical nature, it exhibits insensitivity polarization with absorption peaks of 98.5%, 97.7%, 94.8% and 96% at the frequencies 4.34, 6.68, 8.58, and 10.64 GHz, respectively. Barde et al. described an MTM absorber in^15^ which is compact wideband, polarization-insensitive, and it is applicable for Ku- band radar, satellite, and defense. This MMA absorbs the incident wave from 11.39 to 20.15 GHz with a bandwidth of 8.76 GHz, which completely covers the Ku-band. The dimension of this MMA is 10 × 10 mm^2^. Barde et al. presented another MTM absorber in^16^; it is a set square shape and polarization insensitive. The dimension, FWHM bandwidth, and center frequency of this MMA are 10 × 10 mm^2^, 3.55 GHz, and 9.61 GHz, respectively. Shahidul et al. presented an MMA which shape is hexagonal gap coupled^17^. The peaks of absorption are 99%, 98%, and 81% at the 4.27, 5.42, and 12.40 GHz frequency, respectively, and it covers C- and Ku- bands. Wang Xin et al. demonstrated a dual-band flexible MTM absorber in^18^, which shows the peaks of the absorption 98.7%, 99.3% at the resonance frequency 16.77 GHz and 30.92 GHz, respectively. Yuan et al. presented a geometric-phase metasurface in^19^ for controlling wavefronts of circular polarized (CP) EM waves, which are severely restricted to the cross-polarization modality. The phase addressing mechanism leads to developing new components, such as achromatic broadband devices and wavefront multiplexing for use in reconfigurable-beam antenna and wireless communication systems. An asymmetric circular SRR quad-band MMA is presented in^20^ for the application of multiband. The peaks of absorption are 97.9%, 99.1%, 99.5%, and 99.95% at 4.1, 6.86, 11.3, and 13.45 GHz, respectively. The size and EMR of the MMA are 8 × 8 mm^2^, 9.15 and covering bands are C-, X-, and Ku- band. Sarkhel et al. have proposed a quad-band metamaterial absorber which is compact and polarization-insensitive in^21^. This MMA exhibits several absorption peaks 96.15%, 99.17%, 99.75% and 98.75% at the resonance frequency 3.68, 8.58, 10.17, and 14.93 GHz correspondingly. 10 × 10 mm^2^ are the dimension of this MMA unit cell. Hoque et al. demonstrated a polarization independent MMA for the application of sensing in^22^. The peaks of absorption of this absorber are 99.6% and 99.14% at the frequency 13.78 and 15.3 GHz correspondingly, which cover the Ku- band. The size and EMR of the absorber are 20 × 20 mm^2^ and 1.09, respectively. Ranjan et al. demonstrated a novel binary wind optimization (WDO) algorithm to synthesize six-band MMA in^23^. The dimension of this unit cell is 16 × 16 mm^2^. The absorption peaks of the MMA are more than 90% at the resonance frequencies 7.6, 10.1, 13.3, 14.7, 15.8, and 16.6 GHz. Ranjan et al. also illustrated a novel ultrathin five-band MMA in^24^ which is polarization insensitive. The absorption peaks are 88.99, 94.45, 87.58, 93.06, and 90.42% at the resonance frequencies 2.7, 6.9, 7.3, 13.6, and 16.9 GHz. Agarwal et al. proposed an X-band MMA with dual-band peaks of absorption 95.16% at 8.70 GHz and 97.84% at 10.64 GHz in^25^. In recent times, one MMA is demonstrated for two bands application X-, and Ku- bands in^26^, the absorption peaks can be seen 94.63% at 11.31 GHz, 95.58% at14.11 GHz, 97% at 14.23 GHz, and 75.58% at 17.79 GHz. A novel DNG MMA presented in^27^, this paper shows the Ku-and K-bands absorption. The highest absorption peak shows of 93.72% at the Ku-band and 94.43% at the K-band for the satellite communication. The dimension of this MMA unit cell is 10 × 10 mm^2^. Zhang et al. were presented a noninterleaved polarization-engineered metasurface in^28^ for integer and fractional OAM (orbital angular momentum multiplexing). Pancharatnam–Berry (PB) phase is a direct resource for generating phase gradient along the azimuthal direction required by specific OAM modes for circular polarization incidence. One of the most significant disadvantages of the PB phase is that it only affects cross-polarized fields, leaving the co-polarized portion unmodulated. Mishra et al. demonstrated a polarization-independent MMA, which shows a triple band that is compact ultra-thin^29^. Absorption peaks of this MMA are around 99.67%, 99.48% and 99.42% at the frequencies 4.19, 9.34, and 11.48 GHz, respectively. The size of this MMA unit cell is 8 × 8 mm^2^. Yuan et al. demonstrated a metasurface in^30^ as an energy-controllable circular polarization router that is fully phase-modulated. Spin-selective optics, spin Hall-metasurface, and multi-tasked metasurfaces working in both reflective and transmissive modes are expected to have a wide range of applications. Hoa1 et al. introduced a broadband MMA in^31^ that show at the C-band frequency range absorptivity above 90%. The dimension of this MMA is 15.6 × 15.6 mm^2^. Ranjan et al. presented a novel binary WDO (BWDO) in^32^ that can be specifically used to solve binary-valued problems like antenna array and metasurface synthesis. The unit cell size is 16 × 16 mm^2^.

In this paper, we have proposed an SNG MAA for the multiband applications. The recommended MAA includes the S-, C-, X- and Ku- bands. The frequency range of the S-band is (2–4) GHz, and this band is used for mobile communications, mobile satellite communications and weather ship radar. The frequency range (4–8) GHz known as C-band, application of this band is satellite communication, Wi-Fi devices and weather radar system. X-band (8−12 GHz) is used for radar applications for weather monitoring used in various communication devices. Besides, Ku-band (12–18 GHz) is used in satellite and radar applications also fire detection and sensing radars. Using MATLAB tools, metamaterial properties were extracted, and the absorption percentage was determined. The novelties of this MAA are the simple design, compact size, covers maximum S- band, C- band, X- band, and Ku- band where the absorption peaks of 97.87%, 93.65%, 92.66%, 99.95%, and 99.86% at the frequencies 3.80 GHz, 5.65 GHz, 8.45 GHz, 10.82 GHz, and 15.92 GHz, respectively. The designed MAA unit cell provides high EMR, high quality factor and maximum absorption bands, moreover its size is small compared with many published works^11,13,14,20–22,26,27,29,31^. In order to explain these innovations, this article offers a complete analysis of meta-atom properties of the formed absorber, accompanied by a study of surface current, E and H fields.

## Meta atom unit cell design structure in numerical environment

**Figure 1:**
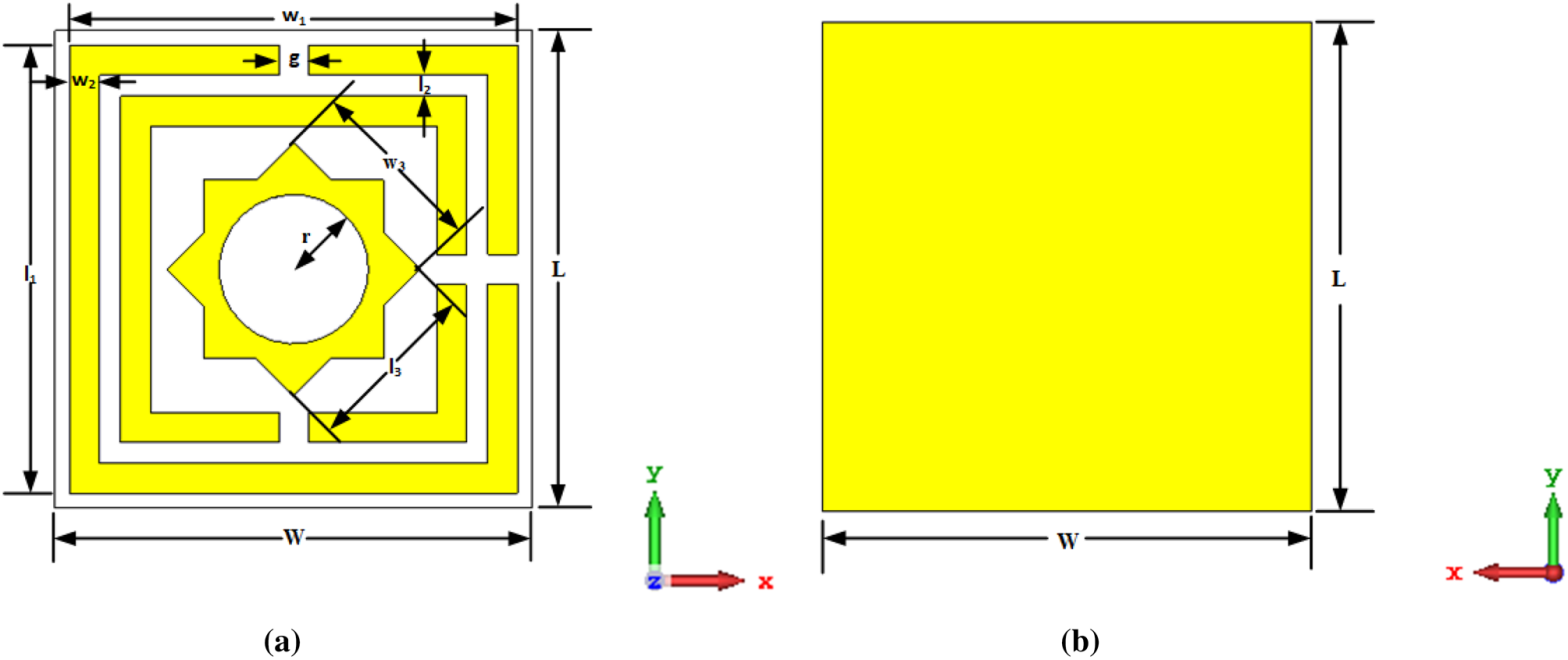
SES-MAA unit cell (**a**) top view with various dimension (**b**) back view with full copper.

Figure 1a,b depicts the SES-MAA unit cell's basic layout with the required dimensions. To construct the MAA unit cell, we have used a minimal-price material FR-4 as a substrate which has a thickness of 1.5 mm, dielectric constant 4.3, and loss factor 0.025. There is a 0.035 mm thickness of copper on both sides of the substrate. Two square SRR with various dimensions are intended at one side of the substrate, and another side is full copper. Each square ring's width is 0.50 mm, and the gap of the split is 0.50 mm. Each square ring has been divided into two parts by the split gap. One-star shape resonator has been placed inside the split square ring. The various dimensions of the suggested MAA unit cell are shown in Table 1.

**Table 1:** Designed MAA unit cell’s parameter value.

Parameters	Size (mm)	Parameters	Size (mm)
*W*	8	*l*_*1*_	7.5
*L*	8	*l*_*2*_	0.35
*w*_*1*_	7.5	*l*_*3*_	3
*w*_*2*_	0.5	*r*	1.25
*w*_*3*_	3	*g*	0.5

**Figure 2:**
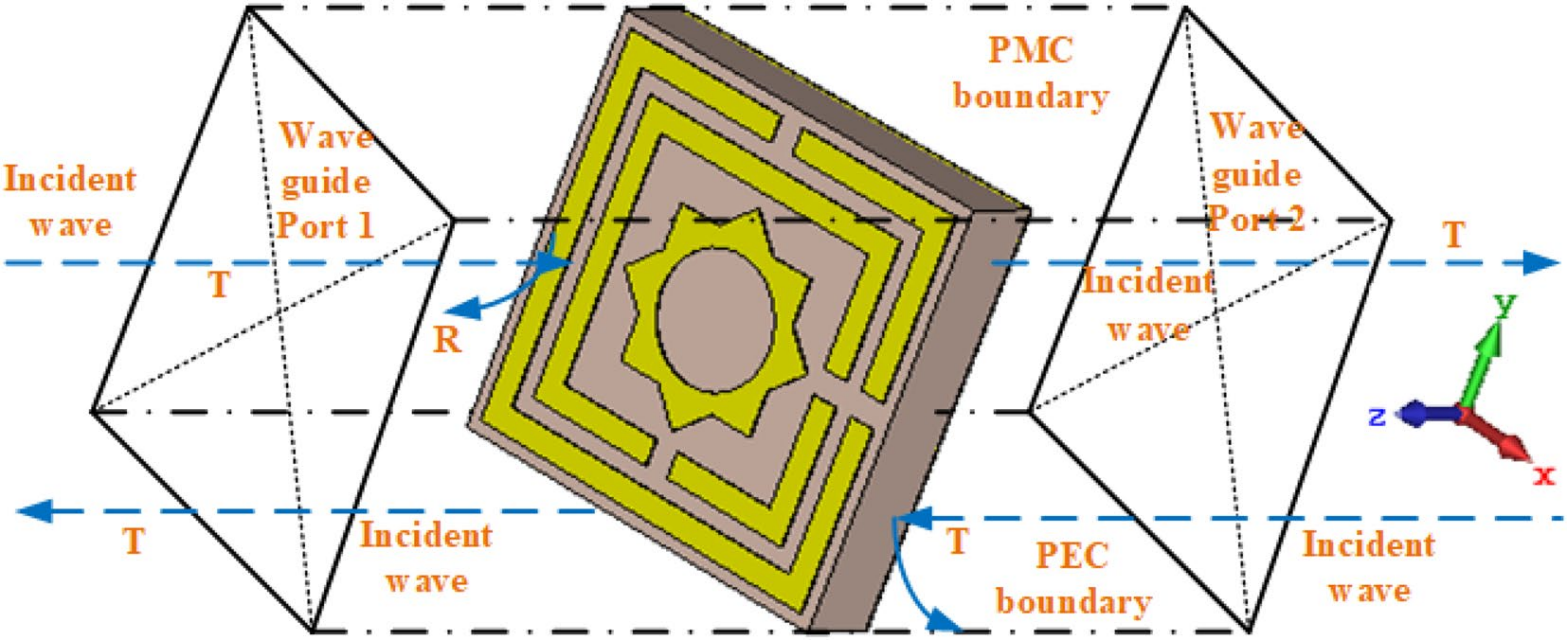
Boundary condition on MAA.

The recommended MAA unit cell's simulation procedure is exhibited in Fig. 2, where a normal incident EM wave is applied in the z-axis, a perfectly electric boundary condition (PEC) is applied in the x-axis, and a perfectly magnetic boundary condition (PMC) is applied in the y-axis. A finite element method (FEM) is used to design the proposed MAA's unit cell. The simulation procedure is done in widely used microwave studio suite CST-2019 software^33^ where 2–18 GHz frequency range has been used. EM wave sources are applied from port 1 to all modes.

## Absorption characteristics investigation of the suggested meta atom absorber unit cell

The absorption $A(f)$, reflection $R(f)$, and transmission $T(f)$ coefficients dependent on frequency are introduced to characterize the MAA unit cell. The relationships among these coefficients are given as^34,35^.

1 $$A(f) = 1 - T(f) - R(f) = 1 - |S_{21} |^{2} - |S_{11} |^{2}$$

With the aim of increase absorptivity $A(f)$, we could decrease the transmission $T(f) = |S_{21} |^{2}$ and the reflection $R(f) = |S_{11} |^{2}$ at the same time. The absorptivity could be estimated by $A(f) = 1 - R(f)$ because the metallic plate blocked the proposed MAA on the bottom layer (So $T(f) = |S_{21} |^{2} = 0$). Hence, the absorptivity of the recommended MAA could be estimated by

2 $$A(f) = 1 - R(f) = 1 - |S_{11} |^{2}$$

It is seen from Eq. (2) that the absorption will be near 100% ($A(f) \approx 100\%$) while the reflectivity is close to zero $(R(f) \approx 0)$. It is noticeable that the $S_{11}$ components include the reflection of co-polarized EM waves and the reflection cross-polarized EM waves ^36,37^. So, the $S_{11}$ components can be expressed as:

3 $$|S_{11} |^{2} = |S_{11,xx} |^{2} + |S_{11,xy} |^{2}$$

Accordingly, based on Eq. (3), Eq. (2) could be evaluated by

4 $$A(f) = 1 - R(f) = 1 - |S_{11,xx} |^{2} - |S_{11,xy} |^{2}$$

Here the $xx$ is co-polarization and $xy$ is the cross-polarization. In the suggested MAA design, $|S_{11} |$ encompasses the components of the co-polarization and cross-polarization.

**Figure 3:**
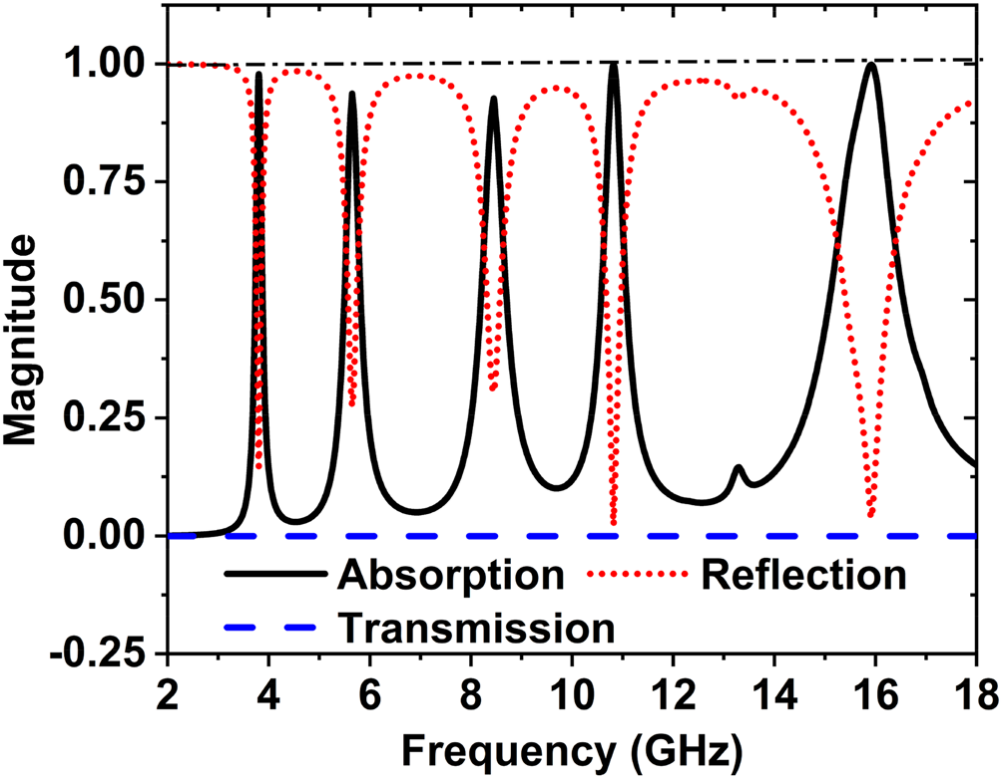
Absorption, reflection, and transmission coefficient of MAA unit cell.

The reflection coefficient $(S_{11} )$, transmission response $(S_{21} )$**,** and absorptance $A(f)$ of the suggested MAA unit cell are shown in Fig. 3. By monitoring this curve, it is found that several absorption peaks occur at 3.80, 5.65, 8.45, 10.82, and 15.92 GHz, along with maximum peaks of absorption 97.87%, 93.65%, 92.66%, 99.95%, and 99.86%, respectively.

**Figure 4:**
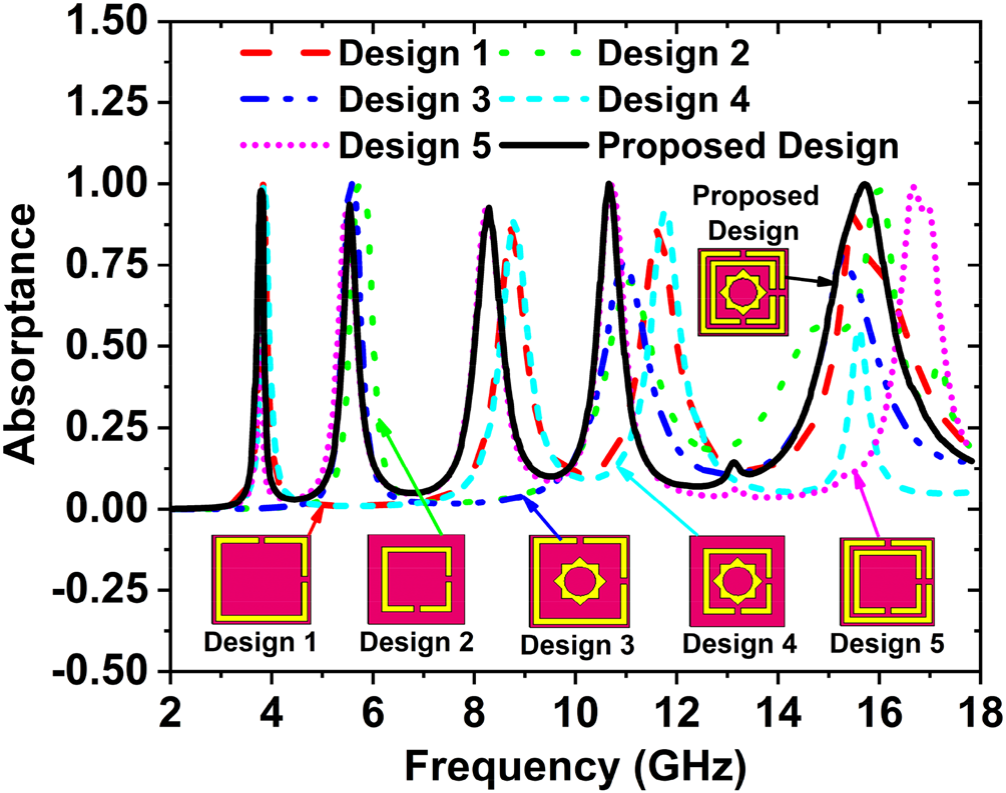
Change of absorption and resonance frequency due to the different design steps.

Figure 4 depicts the change of absorption and resonance frequency due to the different design steps. The final design has been obtained after several steps. When the unit cell contains one large square split ring resonator comprising a width of 0.5 mm, four absorption peaks are available. The absorption peaks are 99.99%, 86.09%, 85.87%, and 92.58% at the resonance frequencies 3.83, 8.74, 11.62, and 15.50 GHz, respectively, denoted by the red dash line in Fig. 4. In design 2 of Fig. 4, one small square SRR exist in the unit cell, and the absorption peaks are 99.67%, 69.92%, and 98.05% at the resonance frequencies 5.76, 11.08, and 15.98 GHz, respectively denoted by the green dotted line. In design 3, the unit cell contains one large square SRR enclosed star, the cell gives three absorption peaks 99.89%, 76.01%, and 77.82% at the resonance frequency 5.57, 10.96, and 15.23 GHz, respectively denoted by a blue dash-dot line. In design 4, the unit cell consists of one small square SRR and one star; this design delivers four absorption peaks 99.99%, 88.46%, 91.91%, and 55.45% at the resonance frequencies of 3.84, 8.78, 11.77, and 15.60 GHz, respectively denoted by the cyan dash line. In the design 5, there are two SRR for the unit cell. The absorption peaks 97.12%, 91.63%, 92.91%, 99.55%, and 98.98% at the resonance frequencies of 3.78, 5.49, 8.26, 10.69, and 16.67 GHz, respectively denoted by a short dot magenta line.

The proposed design consists of two square SRR enclosed with one star. The resonance frequencies are 3.80, 5.65, 8.45, 10.82, and 15.92 GHz, where the absorption peaks are 97.87%, 93.65%, 92.67%, 99.95%, and 99.86%, respectively denoted by the solid black line in Fig. 4. The absorption property is regulated by the distance between two metallic particles, either horizontally or vertically. The absorption efficiency is enhanced by the interaction and coupling between localized surface plasmon (LSP) modes. Different absorption achieved by varying the size and shape of the metallic patch on the substrate is shown in Fig. 4. The two square SRR enclosed one star shape resonator provides high capacitance and inductance, creating perfect impedance matching and effective permittivity and effective permeability. The absorption frequency can also be tunable by changing the geometrical parameters based on L-C resonance circuit theory. Due to the perfect impedance matching and effective permittivity, effective permeability high absorption peaks are achieved, i.e., the absorption peaks are 97.87%, 93.65%, 92.67%, 99.95%, and 99.86% at 3.80, 5.65, 8.45, 10.82, and 15.92 GHz resonance frequencies, respectively. The summarize of the resonance frequency, maximum absorption, and covering bands are listed in Table 2.

**Table 2:** Various design steps with related parameters.

Design phases	Maximum absorption at the frequency (GHz)	Top absorption peaks (%)	Covering bands
Design 1	3.83, 8.74, 11.62, 15.50	99.99, 86.09, 85.87, 92.58	S-, C-, X-, and Ku-
Design 2	5.76, 11.08, 15.98	99.67, 69.92%, 98.05	C-, X-, and Ku-
Design 3	5.57, 10.96, 15.23	99.89, 76.01, 77.82	C-, X-, and Ku-
Design 4	3.84, 8.78, 11.77,15.60	99.99, 88.46, 91.91, 55.45	S-, X-,and Ku-
Design 5	3.78, 5.49, 8.26, 10.69, 16.67	97.12, 91.63, 92.91, 99.55, 98.98	S-, C-, X-, and Ku-
Proposed MAA	3.80, 5.65, 8.45, 10.82, 15.92	97.87, 93.65, 92.67, 99.95, 99.86	S-, C-, X-, and Ku-

The absorber's frequency selectivity is achieved as it provides a narrow bandwidth with a high absorption level in five selected frequencies of 3.80 GHz, 5.65 GHz, 8.45 GHz, 10.82 GHz, and 15.92 GHz. The half-maximum bandwidth (HMBW) at these five selected frequencies were 124 MHz, 220 MHz, 380 MHz, 520 MHz, and 800 MHz, respectively. The following Eq. determines Q-factor

5 $$Q = f_{0} /HMBW$$

where *f*_*0*_ is the maximum absorption peak point frequency, the Q-factors are 30.41, 25.98, 22.53, 20.92, 19.88, and the average Q-factor 23.95 for the suggested MAA cell. The high discrimination of the recommended unit cell of MAA has the potential applicability for sensing and detecting purposes. The unit cell of MAA, whose bandwidth and Q-factor are shown in Table 3.

**Table 3:** MAA unit cell’s Q factor and bandwidth for various application bands.

Frequency at the maximum peak of absorption (GHz)	Percentage of the highest absorption	HMBW (GHz)	Q-factor
3.80	97.87	0.13	30.41
5.65	93.65	0.24	25.98
8.45	92.67	0.51	22.53
10.82	99.95	0.54	20.92
15.92	99.86	1.34	19.88

**Figure 5:**
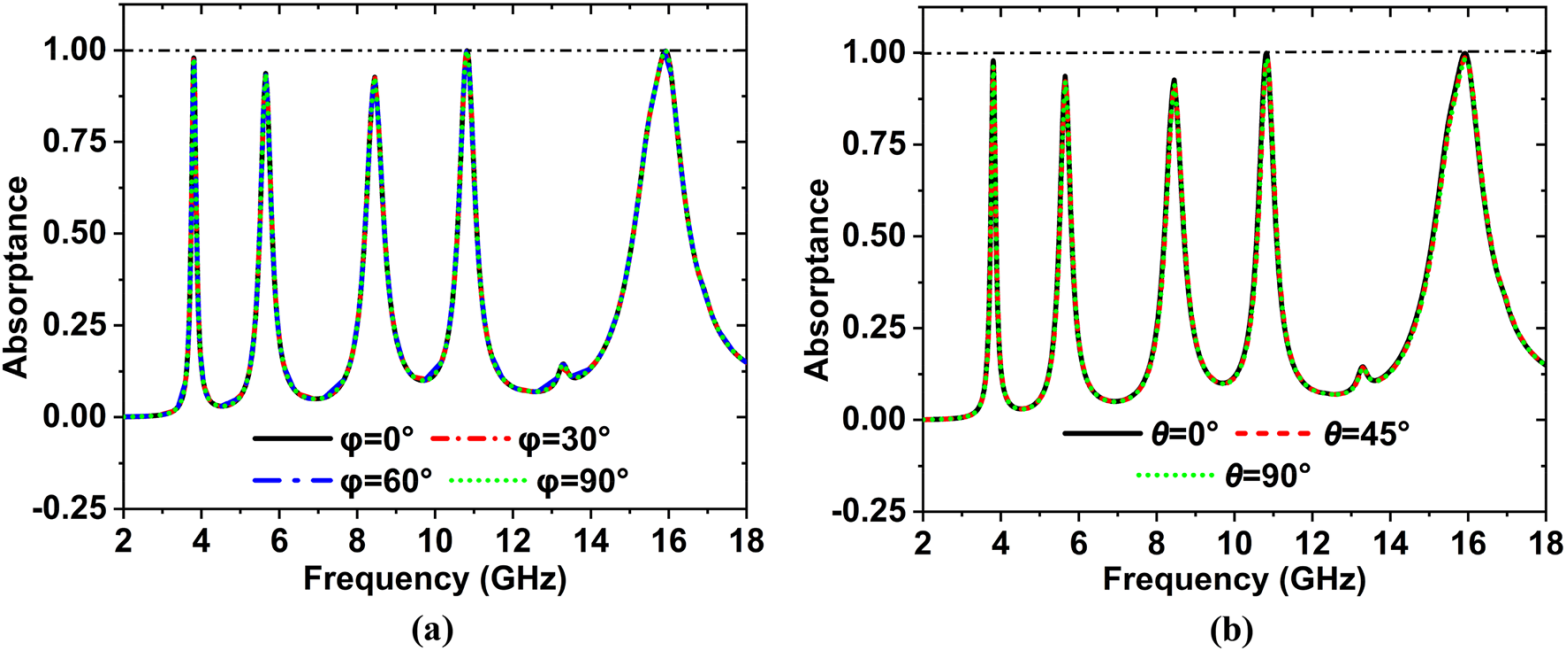
For various polarized EM waves absorptance in TEM mode (**a**) normal incidence (**b**) oblique incidence.

Resonance frequency has been changed very slightly at a higher band, though the absorption remains identical for all polarization angles. The bandwidth of the suggested MAA unit cell's absorption has been accomplished, summing up each resonance peak frequency range with a maximum value of − 10 dB of the corresponding reflection and transmission coefficients. The absorptivity curve for EM wave’s normal incident, the change of polarization angle is presented in Fig. 5a. In this study, E and H fields are rotated at an angle $\varphi$. From this Figure, it is observed that the value of $\varphi = 0^\circ ,{3}0^\circ ,{6}0^\circ ,{9}0^\circ \,$ the absorption coefficient is similar. For the oblique incidence angle $\theta = 0^\circ ,45^\circ ,90^\circ$ the coefficient of absorption is almost the same, which indicates that the proposed MAA unit cell represents polarization-insensitive behavior.

**Figure 6:**
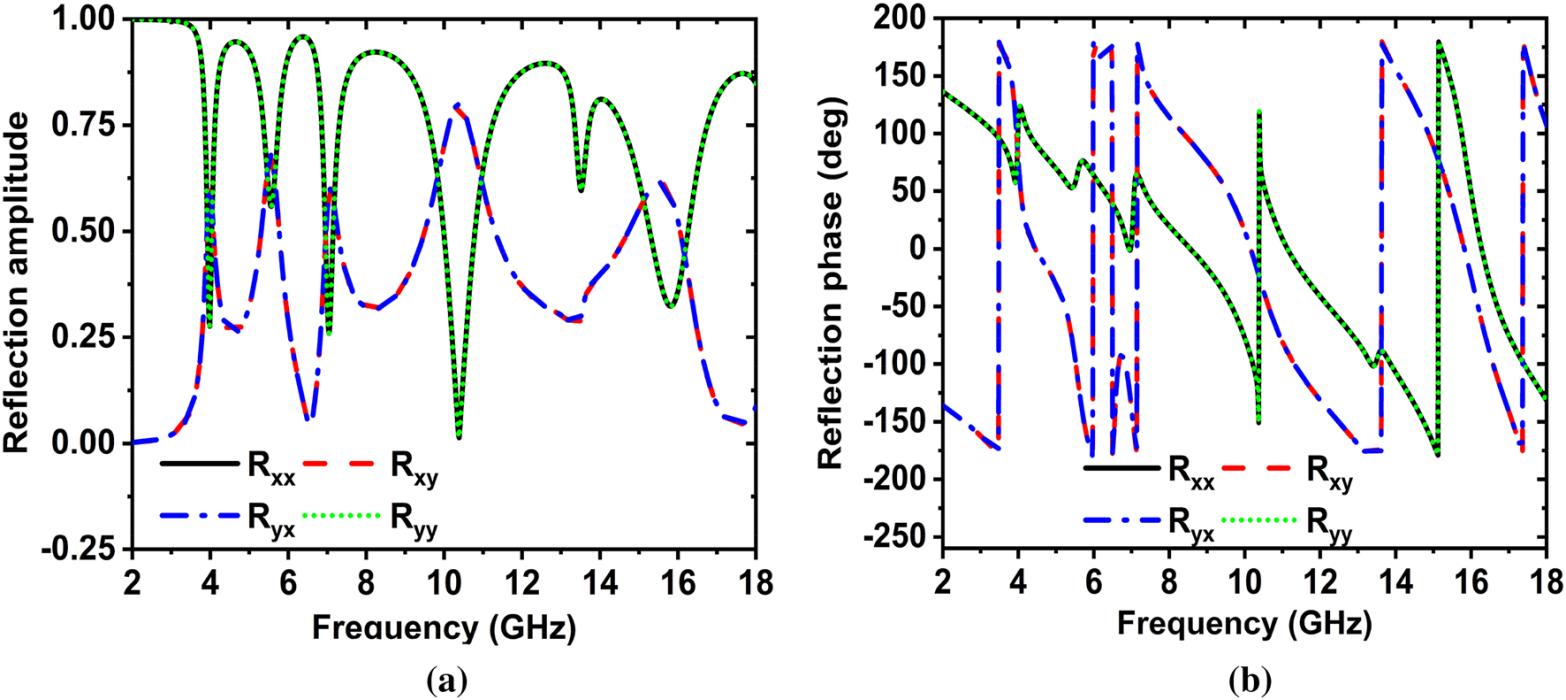
(**a**) Amplitude and (**b**) phase of the reflection response with co-and cross-polarized EM waves.

Figure 5b shows the absorption for the oblique incidence angle $\theta = 0^\circ ,45^\circ ,90^\circ$, respectively, along the x-axis the electric field ($\varphi = 0^\circ$). As the incidence angle increases, the peak absorption value would be almost unchanged^38^. So, the MAA can be achieved near-unity absorbance in a wide range of incidence angles when. Therefore, in a wide variety of incidence angles, the MAA can be accomplished with near-unit absorption when $\varphi = 0^\circ$ and $\theta = 90^\circ$. The amplitude and phase of the reflection response S_11_ with co-and cross-polarized EM waves of the MAA is shown in Fig. 6.

## Meta-atom characteristics of the recommended unit cell

**Figure 7:**
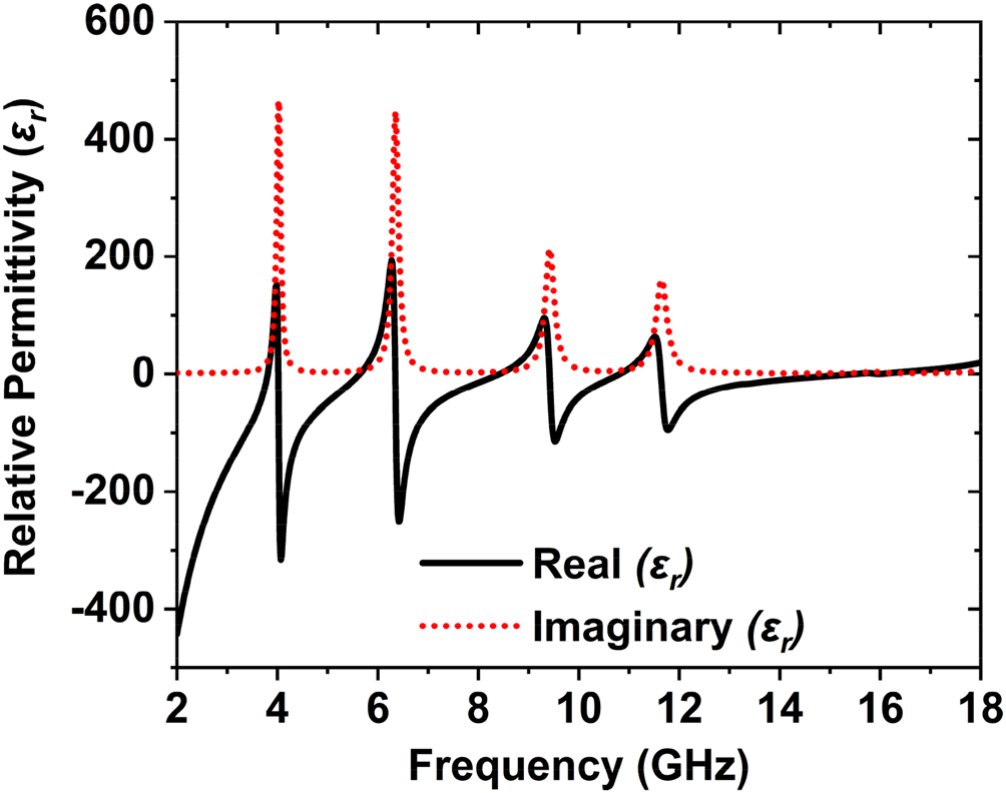
The relative permittivity of the unit cell.

**Figure 8:**
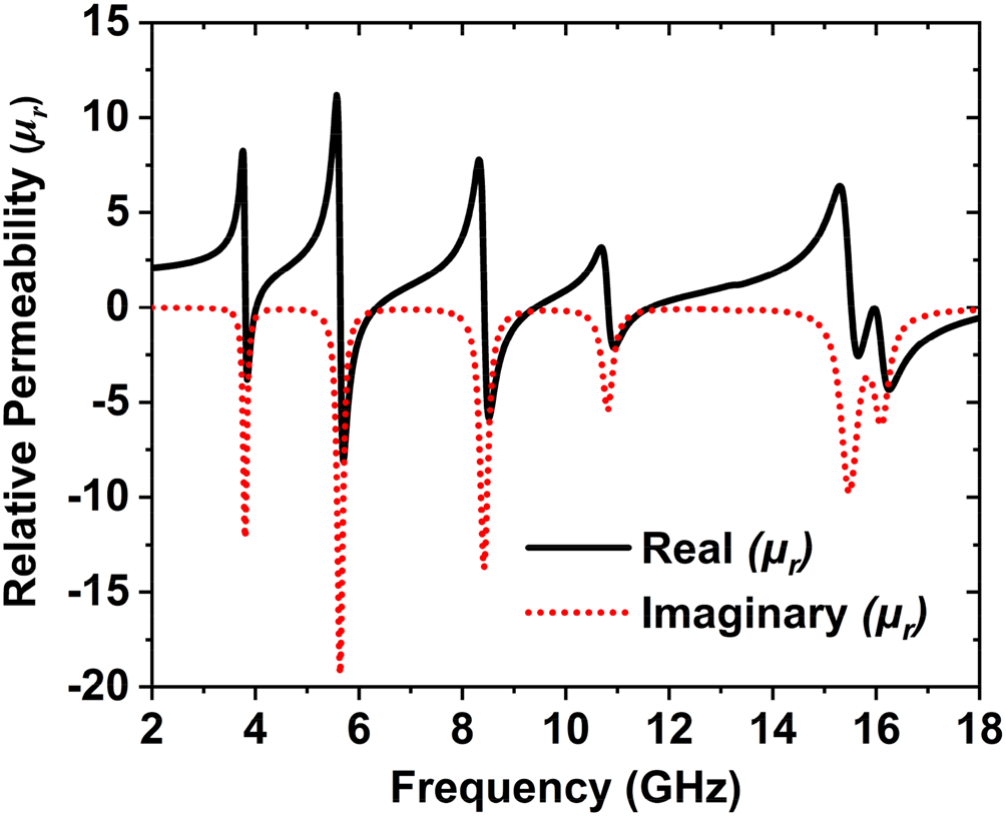
MAA unit cell’s relative permeability.

Meta-atom properties, relative permittivity, and permeability were extracted from the proposed unit cell, as shown in Figs. 7 and 8. Transmission response (S_21_) and reflection response (S_11_) is obtained from the CST simulation^39^ can be used to determine the relative permittivity, relative permeability, and normalized impedance by using Nicholson–Ross–Wier (NRW) approach. If the wave number, $k_{0} = {{2\pi f} \mathord{\left/ {\vphantom {{2\pi f} c}} \right. \kern-\nulldelimiterspace} c}$, where *C* is the speed of light,$f$ is the frequency, then for a substrate having a thickness of $d$, the relative permittivity and relative permeability can be characterized by Eqs. (6), (7)

6 $${\text{Relative}}\;{\text{permittivity}},\;\varepsilon_{r} \sim \frac{2}{{jk_{0} d}} \times \frac{{(1 - S_{11} - S_{21} )}}{{(1 + S_{11} + S_{21} )}}$$

7 $${\text{Relative}}\;{\text{permeability}},\;\mu_{r} \sim \frac{2}{{jk_{0} d}} \times \frac{{(1 - S_{21} + S_{11} )}}{{(1 + S_{21} - S_{11} )}}$$

The following Eqs. can express the refractive index and normalized impedance with the understanding of permittivity and permeability.

8 $${\text{Refractive}}\;{\text{index}},\;n_{r} = \sqrt {\mu_{r} \varepsilon_{r} }$$

9 $${\text{Normalized}}\;{\text{impedance}},\;Z = \sqrt {{{\mu_{r} } \mathord{\left/ {\vphantom {{\mu_{r} } {\varepsilon_{r} }}} \right. \kern-\nulldelimiterspace} {\varepsilon_{r} }}}$$

When permittivity is negative, then the permeability is positive; if the permittivity is positive, the permeability is negative, as shown in Figs. 7 and 8. Table 4 represents the negative region of the relative permittivity, relative permeability, transmission, and reflection coefficient. Table 4 shows that the transmission coefficient around − 200 dB, which is very low. This low value of the transmission coefficient is seen due to the effect of the full copper on the backside, which obstructs the transmission of the EM waves. Thus, the absorption varies only with the coefficient of reflection. The reflection coefficient exhibits below − 10 dB, which is a narrow band. S_11_ shows the five resonances at 3.80, 5.65, 8.45, 10.82, and 15.92 GHz with the reflection peak − 16.72 dB, − 12.19 dB, − 11.13 dB, − 32.70 dB, and 28.49 dB, respectively. These five resonances' bandwidth is 4 MHz, 55 MHz, 60 MHz, 171 MHz, and 449 MHz, respectively. The negative region of the S-parameter and effective parameters are listed in Table 4.

**Table 4:** Effective factors of the recommended MAA unit cell.

Content	Region of frequency (GHz)	Features obtained
Transmission response $(S_{21} )$	18	$S_{21}$ = − 200 dB
Reflection response $(S_{11} )$	3.77–3.82, 5.62–5.68, 8.41–8.47, 10.73–10.90, 15.66–16.10	$S_{11}$ < − 10 dB
Relative permittivity,$\varepsilon_{r}$	2–3.81, 4.01–5.63, 6.35–8.42, 9.42–10.82, 11.63–15.50	$\varepsilon_{r}$ < 0
Relative permeability,$\mu_{r}$	3.81–4, 5.65–6.34, 8.43–9.41, 10.83–11.62, 15.52–18	$\mu_{r}$ < 0

**Figure 9:**
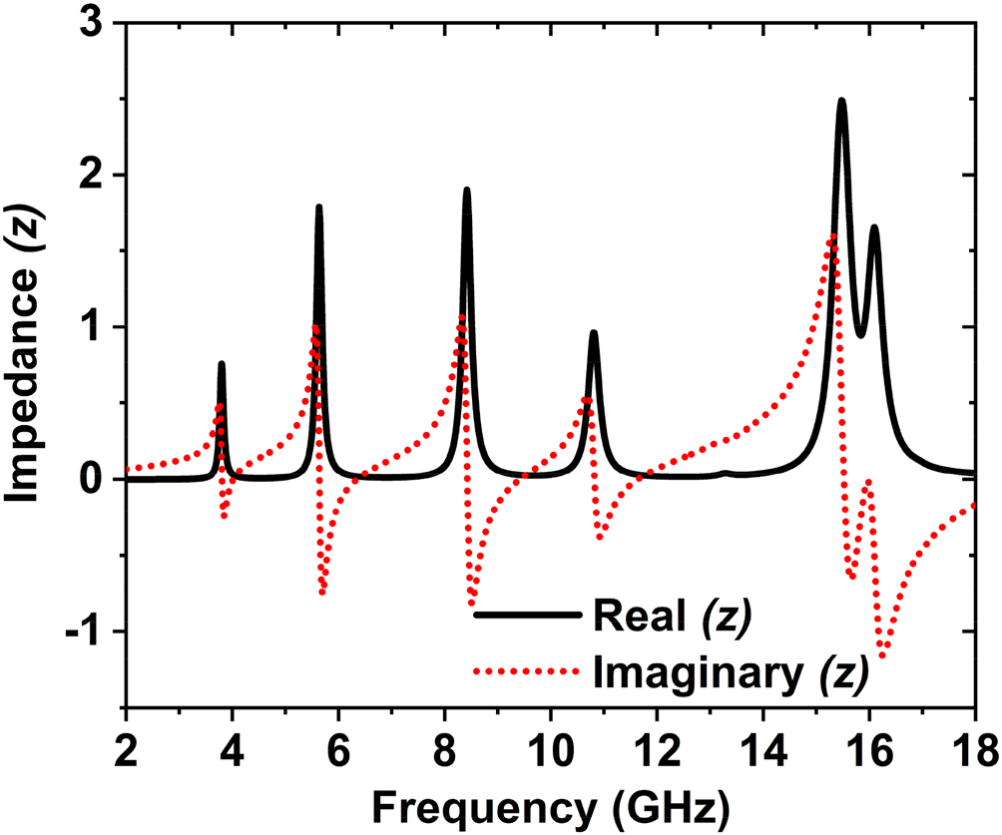
Normalized impedance of the MAA unit cell.

Figure 9 indicates the normalized input impedance. Input impedance has a significant contribution to monitoring the reflection of the incident wave. The meta-atom is classified by the frequency-dependent relative permittivity and permeability to perform the effective medium's characterization. In this incident, the device impedance can be stated as^40^.

10 $$z(\omega ) = \sqrt {\frac{{\mu_{0} \mu_{r} (\omega )}}{{\varepsilon_{0} \varepsilon_{r} (\omega )}}}$$

11 $$Z_{0} = \sqrt {\frac{{\mu_{0} }}{{\varepsilon_{0} }}} = 377ohm$$

where $\varepsilon_{0}$ and $\mu_{0}$ are the free space permittivity and permeability. Thus, zero reflectance can be reached by designing $\varepsilon_{r}$ and $\mu_{r}$ to get them identical from each other. From the impedance graph presented in Fig. 9, it is observed that at all different frequencies real and imaginary part of impedances deviates unity and zero values; thus, absorption is low.

## Surface current, E field and H field distribution of MAA unit cell

**Figure 10:**
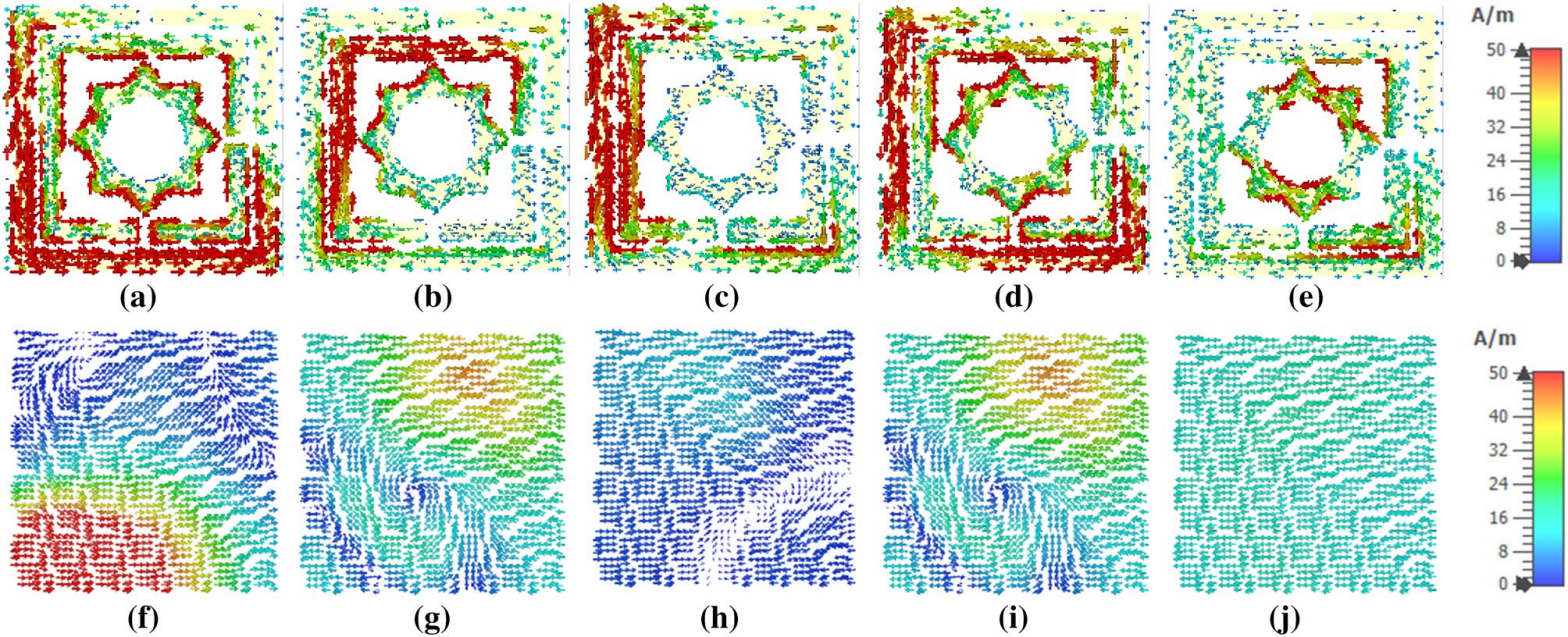
Sufrace current distribution of the MAA unit cell: front view (**a**)–(**e**); back view (**f**)–(**j**) at the resonance frequencies 3.80, 5.65, 8.45, 10.82 and 15.92 GHz.

In order to make explicit, the idea of absorption processes, surface current and electric field analysis be able to perform on the front side and back side of the MAA unit cell device. The surface current forms five major absorption resonances are shown in Fig. 10. At the resonance frequency 3.80 GHz, current flows via the lower and upper left side of two outer rings and the lower right side of the outermost ring. The left segment of the inside ring provides significantly to flow a sufficient quantity of current. The other parts of split rings of horizontal current flows are very small, as depicted in Fig. 10a. The current components of this portion are anti. In the backside, the evenly distributed moderate current flows, which is anti-parallel to the front-end current parallel presented in Fig. 10f. This front and back layer anti-parallel current initiates a current loop, which produces magnetic dipole resonance^41^. At 5.65 GHz, current flows via the left top outermost square split ring and middle ring, as shown in Fig. 10b. The current intensity in the backplane is unevenly dispersed. In the 8.45 GHz absorption peak, a significant current flow through the edges of the left outermost ring, through the top left and bottom left part of two square rings, as revealed in Fig. 10c. It is vital that all over the outer ring, the current density is high. A high dense oppositely flowing current is noticed in the metallic backplane, as shown in Fig. 10h. The current intensity gradually decreases from high to low as it considers the top side. In the top half, and anticlockwise rotating current flows all through the copper. The current circulation shape at 10.82 GHz is shown in Fig. 10d,i for front and backside, respectively. In this frequency, current density becomes low in both front and back. A moderate current flow via the right side of two outer rings. Top coupling point of middle and inner ring, top left corner of outermost ring aids to a significant extent of current. In the backside's metal layer, lower intensity anti-parallel current is noticed that flows from left to right of the structure. This current shows a whirling pattern in the lower right corner. The last absorption peak at 15.92 GHz, where the right bottom portion of the outer two rings and the innermost ring shows the current flow as revealed in Fig. 10e. In the copper backplane significantly lower intensity of current flows is shown in Fig. 10l.

The current distribution, E-and H-fields linked to each other in a time-varying EM wave can be explained by using Maxwell’s curl equations, shown in Eqs. (14) and (15)^42^. Equation (12) describes the EM induction of Faraday’s law, though Eq. (13) is a revised form of Ampere’s law.

12 $$\nabla \times E = - \frac{\partial B}{{\partial t}}$$

13 $$\nabla \times H = J + \frac{\partial D}{{\partial t}}$$

To determine the value of D, the material permittivity ($\varepsilon$) may be applied and for B’s determination, the permeability ($\mu$) can be applied. The connection can be considered by applying Eqs. (14) and (15)

14 $$D(t) = \varepsilon (t) * E(t)$$

15 $$B(t) = \mu (t) * H(t)$$

Take on $e^{j\omega t}$ for time enslavement and substituting time derivative of Eqs. (14) and (15) with $j\omega$, Eqs. (16) and (17) as Maxwell’s curl Eqs. may be stated in phasor

16 $$\nabla \times E = - j\omega \mu H$$

17 $$\nabla \times H = - j\omega \varepsilon E$$

**Figure 11:**
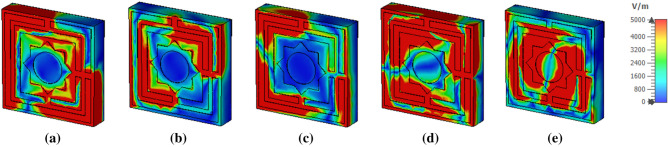
Electric field distribution at (**a**) 3.80 GHz, (**b**) 5.65 GHz, (**c**) 8.45 GHz (**d**) 10.82 GHz and (**e**) 15.92 GHz.

Figure 11a–e represents E field distribution for five absorption peaks at 3.80, 5.65, 8.45, 10.82, and 15.92 GHz, respectively. At 3.80 GHz E field is localized at the left corner of the outer square ring and lower right corner of two square rings. Whereas, at 5.65 GHz, the electric field is observed at the lower-left corner and upper right corner. The right section of the square ring and the split gap of the inner ring represent a powerful electric field. At 8.45 GHz, the medium E-field is noticed at the left low down and upper portion of the two outside square rings and each gap of the split. A major difference of electric field allocation is viewed at 10.82 GHz, wherever a more spreading high E field is noticed near the middle star part of the structure and the left upper part. At 15.92 GHz, a significant electric field is observed in the middle star and left top corner of the square rings. The electric field distribution pattern reveals that as the electric field strength increases with frequency, so does the absorption. This effective induced electric field reverts to the electric field incident, which in turn energizes the electric field than the electric field incident^43^ ensuring electrical resonance. The z-component of the E-field vector by using Maxwell’s Eqs. in free space which can be written as follows:

18 $$\nabla \times \vec{E} = - \frac{1}{c}\frac{{\partial \vec{H}}}{\partial t}$$

19 $$\nabla \times \vec{H} = \frac{1}{c}\frac{{\partial \vec{E}}}{\partial t} + \frac{4\pi }{c}\vec{j}$$

20 $$\nabla \cdot \vec{E} = \rho$$

21 $$\nabla \cdot \vec{H} = 0$$

Taking the curl operation from Eq. (18), we obtain

22 $$\nabla \times (\nabla \times \vec{E}) = \nabla \times \left( { - \frac{1}{c}\frac{{\partial \vec{H}}}{\partial t}} \right)$$

This expression can be simplified as follows:

23 $$\nabla (\nabla \cdot \vec{E}) - \nabla \vec{E} = - \frac{1}{c}\frac{\partial }{\partial t}(\nabla \times \vec{H})$$

After substitution of the corresponding expressions from Eqs. (19) and (20) we derive

24 $$\nabla \rho - \nabla \vec{E} = - \frac{1}{{c^{2} }}\frac{{\partial^{2} \vec{E}}}{{\partial t^{2} }} - \frac{4\pi }{{c^{2} }}\frac{{\partial \vec{j}}}{\partial t}$$

This expression can be re-written in the following way:

25 $$\nabla \vec{E} - \frac{1}{{c^{2} }}\frac{{\partial^{2} \vec{E}}}{{\partial t^{2} }} = \nabla \rho + \frac{4\pi }{{c^{2} }}\frac{{\partial \vec{j}}}{\partial t}$$

To derive the expression for the z-component of the electric field vector, one should project this expression on to the z-axis.

26 $$\nabla E_{z} - \frac{1}{{c^{2} }}\frac{{\partial^{2} E_{z} }}{{\partial t^{2} }} = \frac{\partial \rho }{{\partial z}} + \frac{4\pi }{{c^{2} }}\frac{{\partial j_{z} }}{\partial t}$$

So, the z-component of E-field distributions $(E_{z} )$ of the absolute of E-field distributions $(|E|)$.

**Figure 12:**
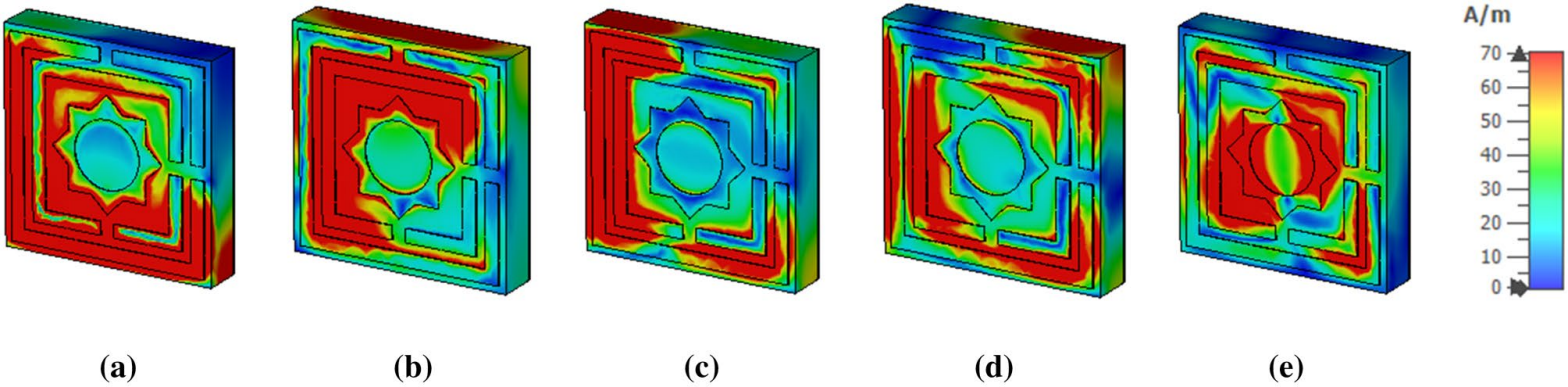
H field distribution of the MAA unit cell at (**a**) 3.80 GHz, (**b**) 5.65 GHz, (**c**) 8.45 GHz (**d**) 10.82 GHz and (**e**) 15.92 GHz.

The magnetic field distribution is presented in Fig. 12a–e for five different absorption peaks at the resonance frequencies. At 3.80 GHz, a sharp magnetic field is observed in the left lower and higher part, the right lower portion, and the MM absorber unit cell's middle star part. At 5.65 GHz, A sharp magnetic field is noticed left part in the upper and lower portion of the middle square and right top part of the star ring. At 8.45 GHz, the magnetic field high for the left is lower to the upper portion of the MAA unit cell. The major amount of H filed is also noticed at 10.82 GHz frequency, as exhibited in Fig. 12d. The H field is circulating on the suggested MAA unit cell. The inner edges of the innermost ring. At 15.92 GHz, the medium H field is seen surrounding the star ring as shown in Fig. 12e.

## Analysis of the MAA unit cell’s equivalent circuit

**Figure 13:**
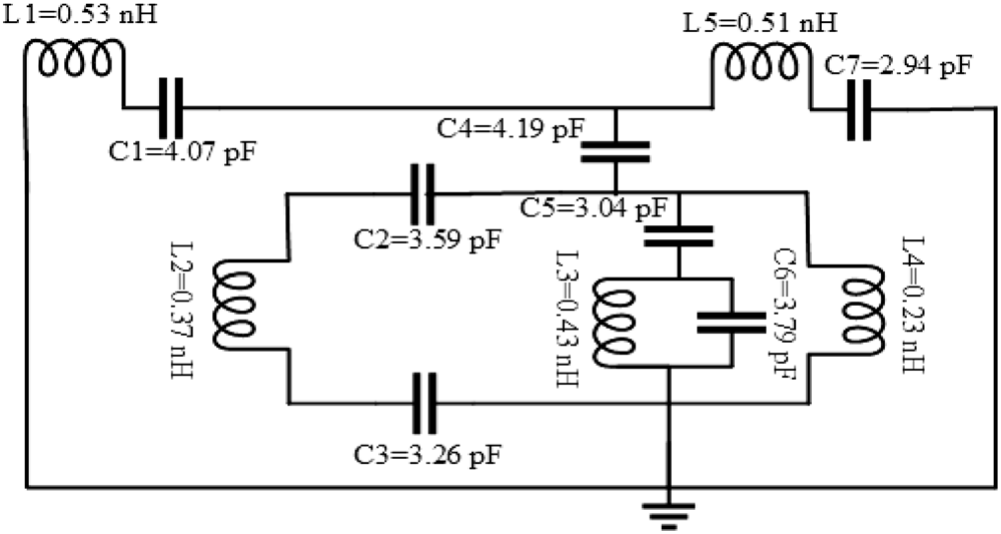
Equivalent circuit modelling of the MAA unit cell.

Figure 13 indicates an estimated circuit for the unit cell. Since the mentioned form involves passive components, i.e., inductive-capacitive *(LC)* elements, the resonance frequency *(f)* is given below^44^.

27 $$f = \frac{1}{{2\pi \sqrt {LC} }}$$

where $L$ represents the collective inductance, and $C$ denotes the collective capacitance. In the designed structure, inductance is formed by the metal strip and assembled of capacitance by the splits. The electrical resonances are created by coupling between the gaps and electric fields. Besides, magnetic resonances are generated by coupling the magnetic fields with the loops. The capacitance

28 $$C = \varepsilon_{0} \varepsilon_{r} \frac{A}{d}(F)$$

where $\varepsilon_{0} = 8.854 \times 10^{ - 12}$ *F/m*, $\varepsilon_{r}$ = relative permittivity, *A* is the split region, and *d* is the split distance. Besides, the total inductance $\left( L \right)$ can be determined by^45^

29 $$L = 0.01 \times \mu_{0} \left\{ {\frac{{2(d + g + h)^{2} }}{{(2w + g + h)^{2} }} + \frac{{\sqrt {(2w + g + h)^{2} + l^{2} } }}{(d + g + h)}} \right\}t$$

where $$\mu_{0} = 4\pi \times 10^{ - 7}$$ H/m.

Therefore, the capacitance $(C)$ can be achieved by

30 $$C = \varepsilon_{0} \left[ {\frac{2w + g + h}{{2\pi (d + h)^{2} }}\ln \left\{ {\frac{2(d + g + h)}{{(a - l)}}} \right\}} \right]t$$

**Figure 14:**
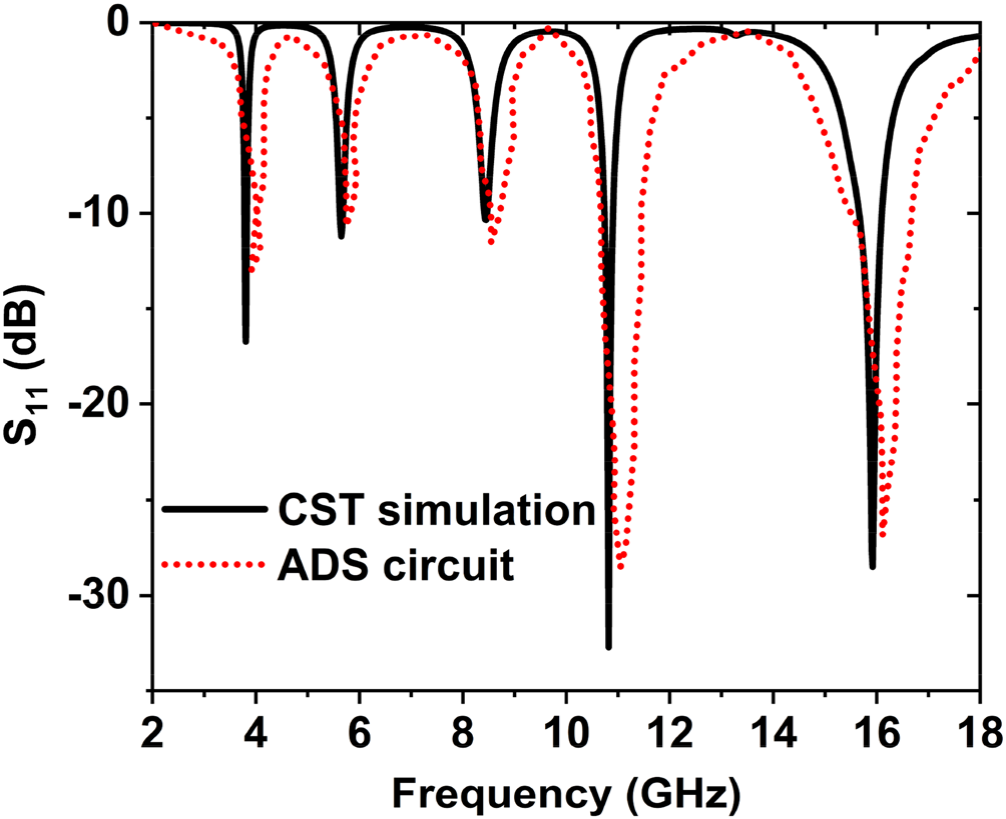
S_11_ result for CST and ADS simulation of MAA.

Microstrip lines are presented with different inductors *L1*, *L2*, *L3*, *L4, L5*, and capacitors are represented by *C1*, *C2*, *C3*, *C4*, *C5*, *C6,* and *C7* due to the split gaps. Because of the two-ring coupling row, *L3* is the inductor, and *C4* is the coupling capacitor. By using advanced design system (ADS) software^46^, the values of these elements are modified. The reflection coefficient is calculated by tuning the parameter values such that the ADS result shows a near resemblance to the outcome obtained from the CST. Figure 14 shows the CST simulated and ADS analysis result of reflection response.

## Analysis of the different array configuration of the MAA

**Figure 15:**
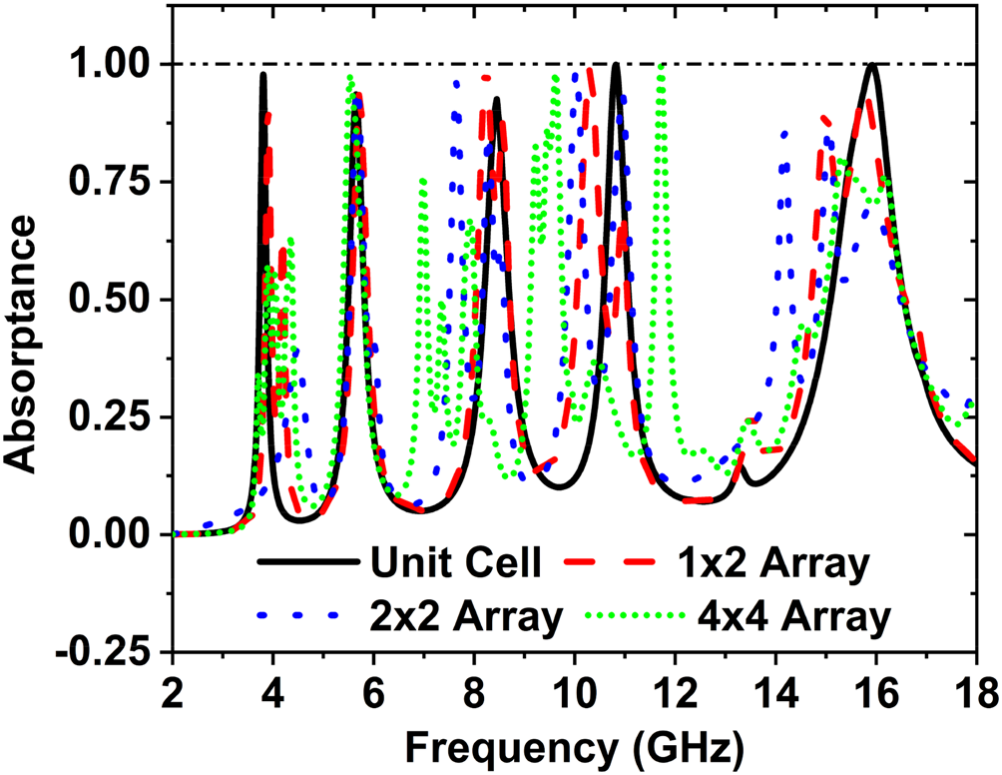
Absorption comparison among different arrays and unit cell.

The unit cell arrays are prepared and simulated to observe the performance of unit cell arrays 1 × 2, 2 × 2, and 4 × 4. The arrays’ absorption is compared to the recommended unit cell, and the absorption plot is revealed in Fig. 15. The EM interaction in the array is complex when the built array is positioned side by side horizontally and vertically. The absorption peaks change magnitude and frequency due to the mutual coupling effect. The Harmonic influence is also evident in the series.

Figure 15 expresses the change of absorption and frequency with the different array configurations. Here to show the effect of absorption peaks and frequencies, we have analyzed 1 × 2 array, 2 × 2 array, and 4 × 4 array. For the unit cell, the obtained absorption peaks are 97.87%, 93.65%, 923.67%, 99.95%, and 99.86% at the frequencies 3.80 GHz, 5.65 GHz, 8.45 GHz, 10.82 GHz, and 15.92 GHz, respectively. The absorption peaks are 91.19%, 94.01%, 99.79%, 98.93%, 94.71% at the frequencies 3.90 GHz, 5.71 GHz, 8.24 GHz, 10.30 GHz, and 94.71 GHz for the 1 × 2 array; 2 × 2 array consists of the 4-unit cell connected as the horizontally and vertically. In this configuration the absorption peaks are 95.51%, 96.25%, 98.98%, 94.18%, 85.50% and 87.06% at the frequencies 5.68, 7.65, 10.03, 10.94, 14.18, and 15.06 GHz, respectively. To construct a 4 × 4 array, 16-unit cells have been used horizontally and vertically. At the 3.89 GHz, 5.54 GHz, 6.98 GHz, 9.62 GHz, 11.71 GHz, 15.32 GHz, the peaks of the absorption are 57.22%, 97.77%, 76.22%, 98.26%, 99.55% and 80.45% respectively. The absorption peaks change in magnitude and shift the frequency due to the effect of mutual coupling. Besides, some harmonics exist in the array due to the mutual coupling effects and construction of the unit cell's asymmetric pattern. The absorber's frequency selectivity is achieved as it provides a narrow bandwidth with a high absorption level in five selected frequencies of 3.80 GHz, 5.65 GHz, 8.45 GHz, 10.82 GHz, and 15.92 GHz. To match the arrays' discrimination, Q-factor has been computed and shown in Table 5 alongside the highest absorptance and frequency of the resonance peak. Table 3 remarked that all the arrays deliver adequate Q-factor within S-, C-, X- and Ku- bands. The results of the arrays are summarized in Table 5.

**Table 5:** Different parameters for various array configuration.

Structure	Resonance frequency (GHz)	Absorption peak (%)	Q factor	Frequency GHz)	Absorption peak (%)	Q factor
Unit cell	3.80	97.87	30.41	10.82	99.94	20.92
	5.65	93.65	25.98	15.92	99.85	19.88
	8.45	92.66	22.53	–	–	–
1 × 2 array	3.90	91.19	29.74	10.34	98.93	17.36
	5.71	94.04	23.23	15.76	94.71	11.43
	8.24	99.79	20.27	–	–	–
2 × 2 array	5.68	95.51	27.39	14.18	85.50	24.52
	7.65	96.25	21.53	15.06	87.06	13.63
	10.03	98.98	6.5	–	–	–
4 × 4 array	3.89	57.22	26.51	9.62	98.26	24.74
	5.54	97.77	24.43	11.71	99.55	23.67
	6.98	76.23	27.16	15.32	80.45	14.18

## Parametric analysis

### Effect of the copper backplane

**Figure 16:**
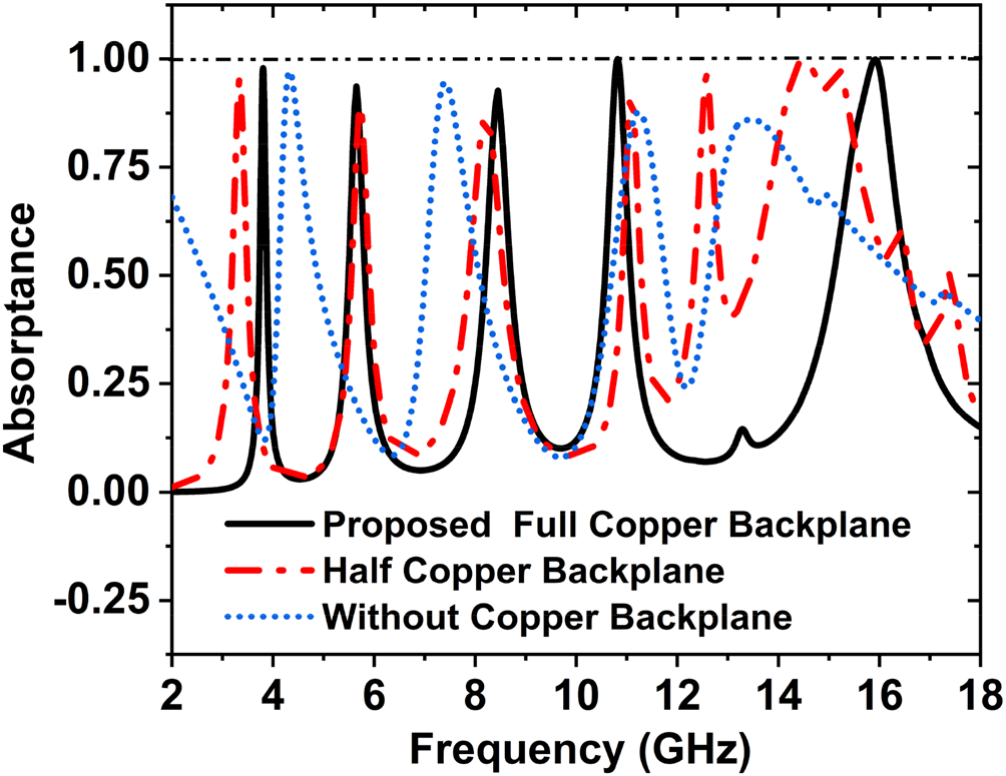
Change of absorption with the variation of the copper backplane.

Figure 16 illustrates the effect of different sizes of copper backplane on the absorption peaks and frequencies. To show the impact on absorption with the different sizes of the copper backplane, we have used a full copper backplane, half copper backplane, and without copper backplane. When the copper has been used as a full backplane, the peaks of the absorption are 97.87%, 93.65%. 92.67%, 99.95%, and 99.86% at the frequencies 3.80, 5.65, 8.45, 10.82, and 15.92.

GHz, respectively. If the copper is used as a half backplane, 95.42%, 89.23%, 87.67%, 92.64%, 96.07%, and 99.72% absorption peaks are obtainable at the 3.34 GHz, 5.72 GHz, 8.21 GHz, 11.08, 12.58, and 14.49 GHz frequencies, respectively. At the frequencies 4.32, 7.41, 11.23, and 13.33 GHz, the absorption peaks are 97.24%, 94.66%, 87.92%, and 86.09%, respectively for without copper backplane. Since a full copper backplane shows the best performance, so full copper backplane has been used for the suggested MAA unit cell. The response of the backplane on the resonance frequency and absorption peaks are listed in Table 6.

**Table 6:** Effect of copper backplane on absorption peaks and frequencies.

Copper backplane	Resonance frequency (GHz)	Maximum absorption (%)	Covering bands
Without	4.32, 7.41, 11.23, 13.33	97.24, 94.66, 87.92, 86.09	C-, X-, and Ku-
Half	3.34, 5.72, 8.21, 11.08, 12.58, 14.49	95.42, 89.23, 87.67, 92.64, 96.07, 99.72	S-, C-, X-, and Ku-
Proposed full	3.80, 5.65, 8.45, 10.82, 15.92	97.87, 93.65, 92.67, 99.95, 99.86	S-, C-, X-, and Ku-

### Effect of the substrate size

**Figure 17:**
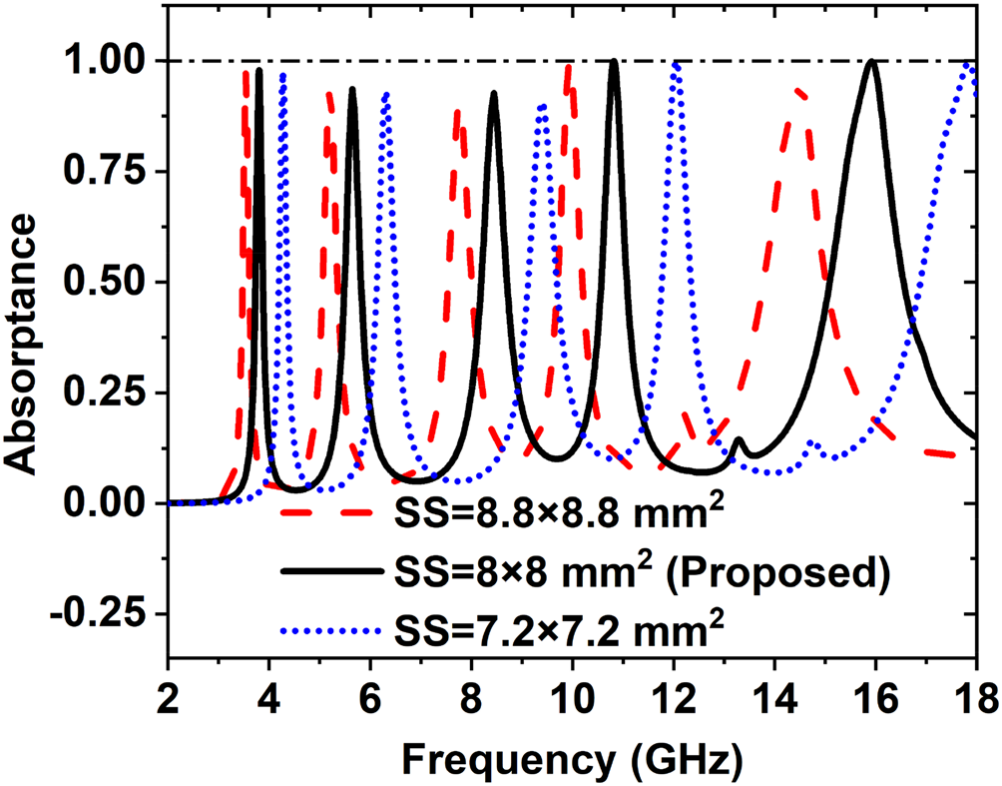
Effect of size of the substrate on the absorption peaks and resonance frequency.

We have taken three sizes of the substrate to see the effect of absorption peak and resonance frequency. The substrate’s three dimensions are 8.8 × 8.8 mm^2^, 8 × 8 mm^2^, and 7.2 × 7.2 mm^2^. When we have taken the substrate size 8.8 × 8.8 mm^2^, the five peak points are obtained; these are 97.06%, 93.47%, 90.25%, 99.81%, and 95.32% at the resonance frequencies of 3.54, 5.20, 7.76, 9.95, and 14.53 GHz, respectively. If the substrate size is 8 × 8 mm^2^, the absorption peaks are 97.87, 93.65, 92.67, 99.95, 99.86 at the frequencies of 3.80, 5.65, 8.45, 10.82, and 15.92 GHz, respectively. If the substrate's size is 7.2 × 7.2 mm^2^ then the resonance frequencies 4.27, 6.30, 9.39, 12.05, and 17.84, showing the absorption peaks 97.49%, 93.09%, 90.67%, 99.97%, and 99.52% respectively. Figure 17 shows the effect of different size of the substrate on the absorption peaks and the resonance frequency. Table 7 depicts the effect of substrate size on absorption peaks, resonance frequencies, and covering bands.

**Table 7:** Effect of substrate size on absorption peaks, resonance frequencies, and covering bands.

Substrate size (mm^2^)	Resonance frequency (GHz)	Maximum absorption (%)	Covering bands
8.8 × 8.8	3.54, 5.20, 7.76, 9.95, 14.53	97.06, 93.47, 90.25, 99.81, 95.32	S-, C-, X-, and Ku-
8 × 8	3.80, 5.65, 8.45, 10.82, 15.92	97.87, 93.65, 92.67, 99.95, 99.86	S-, C-, X-, and Ku-
7.2 × 7.2	4.27, 6.30, 9.39, 12.05, 17.84	97.49, 93.09, 90.67, 99.97, 99.52	C-, X-, and Ku-

From Table 7, it is clearly seen that for substrate size 7.2 × 7.2 mm^2^, the absorption peaks are low and covering band C-, X-, and Ku- whereas the covering band is S-, C-, X-, and Ku- and absorption peaks are high for substrate size 8 × 8 mm^2^. Since the substrate size 8 × 8 mm^2^ covers maximum bands and absorbs high peaks, hence we have considered the substrate size of 8 × 8 mm^2^.

### Effect of the patch size

**Figure 18:**
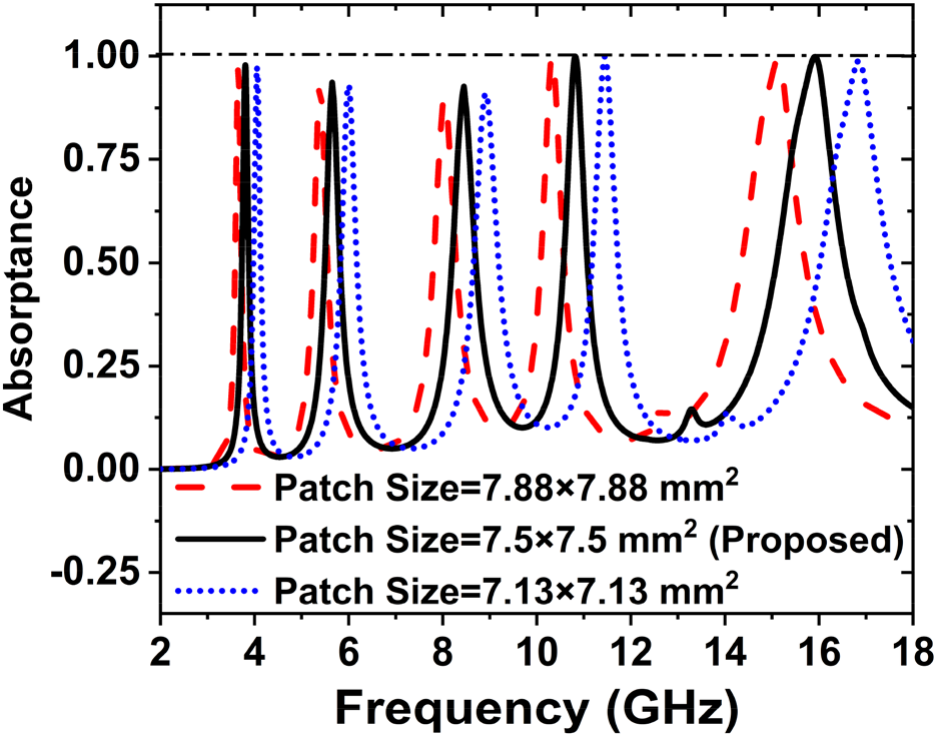
Effect of size of the patch on the absorption peaks and resonance frequency.

Figure 18 shows the effect of different size of the patch on the absorption peaks and resonance frequency. Different size of the patch has been used for the performance analysis of the proposed absorber. The different size of the outer square patches is 7.88, 7.5, and 7.13 mm. If the patch size is 7.88 mm then the peaks of absorption are shows 97.54%, 93.09%, 89.97%, 99.99%, 99.28% at the resonance frequencies of 3.64, 5.39, 8.03, 10.33, 15.12 GHz, respectively. If the patch's size is 7.5 mm, the resonance frequencies 3.80, 5.65, 8.45, 10.82, and 15.92 GHz shows the absorption peaks are 97.87%, 93.65%, 92.67%, 99.95%, and 99.86% respectively. When 7.13 mm size was taken for patch, the absorption peaks were 97.46%, 93.09%, 91.09%, 99.91%, 98.86% shows at the frequencies of 4.05, 6, 8.91, 11.44, and 16.85 GHz, respectively. Table 8 depicts the effect of patch size on absorption peaks, resonance frequencies, and covering bands.

**Table 8:** Effect of patch size on absorption peaks, resonance frequencies, and covering bands.

Patch size (mm^2^)	Resonance frequency (GHz)	Maximum absorption (%)	Covering bands
7.88 × 7.88	3.64, 5.39, 8.03, 10.33, 15.12	97.54, 93.09, 89.97, 99.99, 99.28	S-, C-, X-, and Ku-
7.5 × 7.5	3.80, 5.65, 8.45, 10.82, 15.92	97.87, 93.65, 92.67, 99.95, 99.86	S-, C-, X-, and Ku-
7.13 × 7.13	4.05, 6, 8.91, 11.44, 16.85	97.46, 93.09, 91.09, 99.91, 98.86	C-, X-, and Ku-

From Table 8, it is clearly seen that for patch size 7.13 × 7.13 mm^2^, the absorption peaks are low and covering bands are C-, X-, and Ku- whereas the covering bands are S-, C-, X-, and Ku- and absorption peaks are high for patch size 8 × 8 mm^2^. Since the patch size 7.5 × 7.5 mm^2^ covers maximum band and absorb high peaks, hence we have considered the outer patch size of 7.5 × 7.5 mm^2^.

### Effect of the split gap

**Figure 19:**
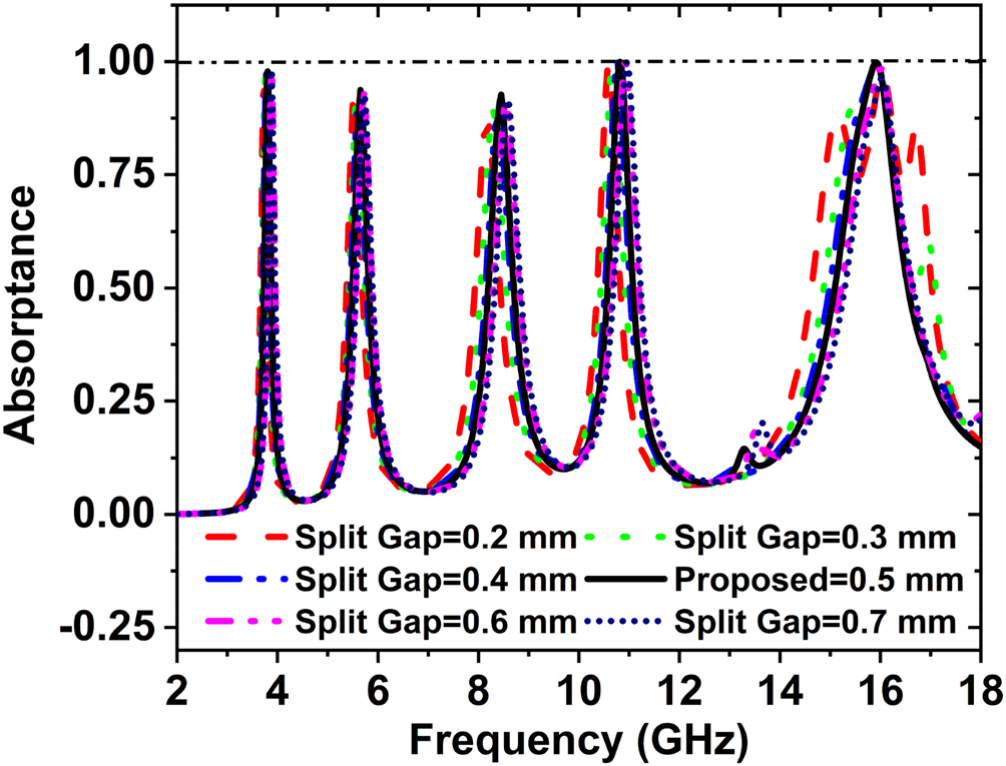
Change of absorption with the variation of the split gap.

Figure 19 indicates the effect of the split gap on absorption peak and resonance frequency. When the split gap is increased, the resonance frequency is also increased because the resonance frequency depends on the capacitor. The capacitor is inversely proportional to the split gap. Also, the capacitor is inversely proportional to the resonance frequency. For split gap 0.2 mm, the absorption peaks are 95.85%, 93.34%, 87.44%, 99.21%, and 88.19% at the resonance frequencies of 3.72, 5.52 8.18, 10.59, and 15.15 GHz, respectively. The absorption peaks 96.70%, 93.16%, 88.95%, 99.81%, and 97.97% at the resonance frequencies of 3.76, 5.58, 8.29, 10.67, and 15.95 GHz, respectively for the split gap 0.3 mm. If the split gap is 0.4 mm, the absorption peaks are 97.59%, 92.39%, 90.22%, 99.82%, and 99.75% at the frequencies of 3.77, 5.61, 8.38, 10.77, and 15.92 GHz, respectively. For split gap 0.5 mm, the absorption peaks are 97.87%, 93.65%, 92.67%, 99.95% and 99.86% at the frequencies 3.80, 5.65, 8.45, 10.82, and 15.92 GHz, respectively.

At the frequency 3.88, 5.71, 8.54, 10.92, and 16.01 GHz, the peaks of the absorption are 97.98%, 93.01%, 91.16%, 99.86%, and 99.59% respectively for the split gap 0.6 mm. The absorption peaks are 98.35%, 92.66%, 91.65%, 99.85%, and 96.28% at the frequencies of 3.88, 5.72, 8.58, 10.96, and 16.03 GHz respectively for 0.7 mm split gap. Since the best performance shows for the split gap of 0.5 mm, the split gap has been selected 0.5 mm for the suggested MAA unit cell.

### Effect of different substrate material

**Figure 20:**
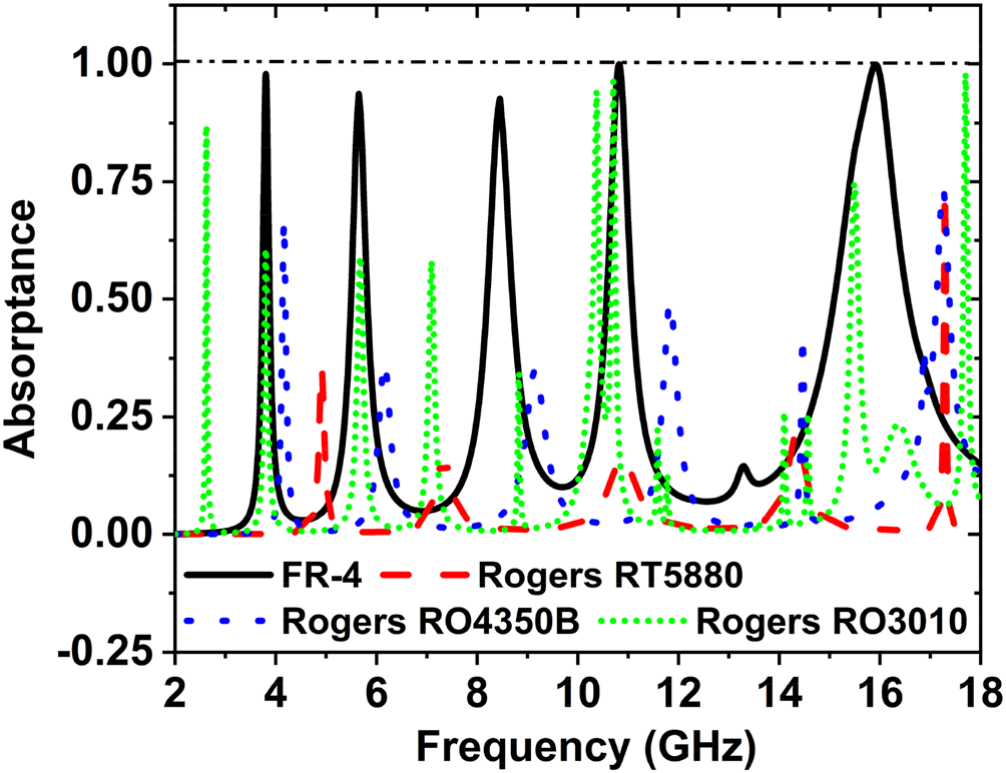
Effect of different substrate material on the absorption peak curve.

Figure 20 indicates the effect of different substrate materials on the absorption peak and frequency. There are four substrate materials used to show the effect of the absorption peaks and frequencies. These materials are FR-4, Rogers RT5880, Rogers RO4350B, and Rogers RO3010. The thickness (t), loss tangent *(LT*), and dielectric constant *(DC)* of FR-4 substrate are 1.5 mm. 0.025 and 4.3, respectively. The thickness, *LT*, and DC of the Rogers RT5880 are 1.575 mm, 0.0009 and 2.2, respectively. 1.524 mm, 3.66, 0.0037 are the thickness, *DC*, and *LT* of Rogers RO4350B.

Rogers RO3010 had a thickness of 1.27 mm, DC 11.2, and *LT* 0.0022. The absorption peaks for the FR-4 substrate are 97.87%, 93.65%, 92.67%, 99.95% and 99.86% at the frequency 3.80, 5.65, 8.45, 10.82, and 15.92 GHz, respectively. At the frequency of 4.91, 7.33, 10.89, 14.34, and 17.29 GHz, the absorption peaks are 35.91%, 16.67%, 16.28%, 21.28%, and 73.52%, respectively, for the Rogers RT5880 substrate material. When the substrate material Rogers RO4350B is used, the peaks of the absorption are 66.94%, 36.67%, 35.15%, 49.43%, 42.67%, and 73.79% at the frequencies of 4.16, 6.17, 9.15, 11.82, 14.64, and 17.248 GHz, respectively. For Rogers RO3010 substrate material, the absorption peaks are 86.97%, 60.75%, 59.12%, 58.14%, 94.28%, 96.42%, 74.66% and 97.99% at the 2.62, 3.81, 5.68, 7.09, 10.37, 10.70, 15.49, and 17.69 GHz frequency, respectively. From this analysis, it is said that the substrate material FR-4 shows the best performance for absorption, so FR-4 is used as the substrate for the recommended design. The performance of the different substrate materials is shown in Table 9.

**Table 9:** Change of absorption peaks and frequencies with the different substrate material.

Substrate material	Resonance frequency (GHz)	Absorption peaks (%)	Covering bands
FR-4	3.80, 5.65, 8.45, 10.82, 15.92	97.87, 93.65, 92.67, 99.95, 99.86	S-, C-, X-, and Ku-
Rogers RT5880	4.91, 7.33, 10.89, 14.34, 17.29	35.91, 16.67, 16.28, 21.28, 73.52	C-, X-, and Ku-
Rogers RO 4350B	4.16, 6.17, 9.15, 11.82, 14.64, 17.248	66.94, 36.67, 35.15, 49.43, 42.67, 73.79	C-, X-, and Ku-
Rogers RO3010	2.62, 3.81, 5.68, 7.09, 10.37, 10.70, 15.49, 17.69	86.97, 60.75, 59.12, 58.14, 94.28, 96.42, 74.66, 97.99	S-, C-, X-, and Ku-

## Measurement results and discussion

**Figure 21:**
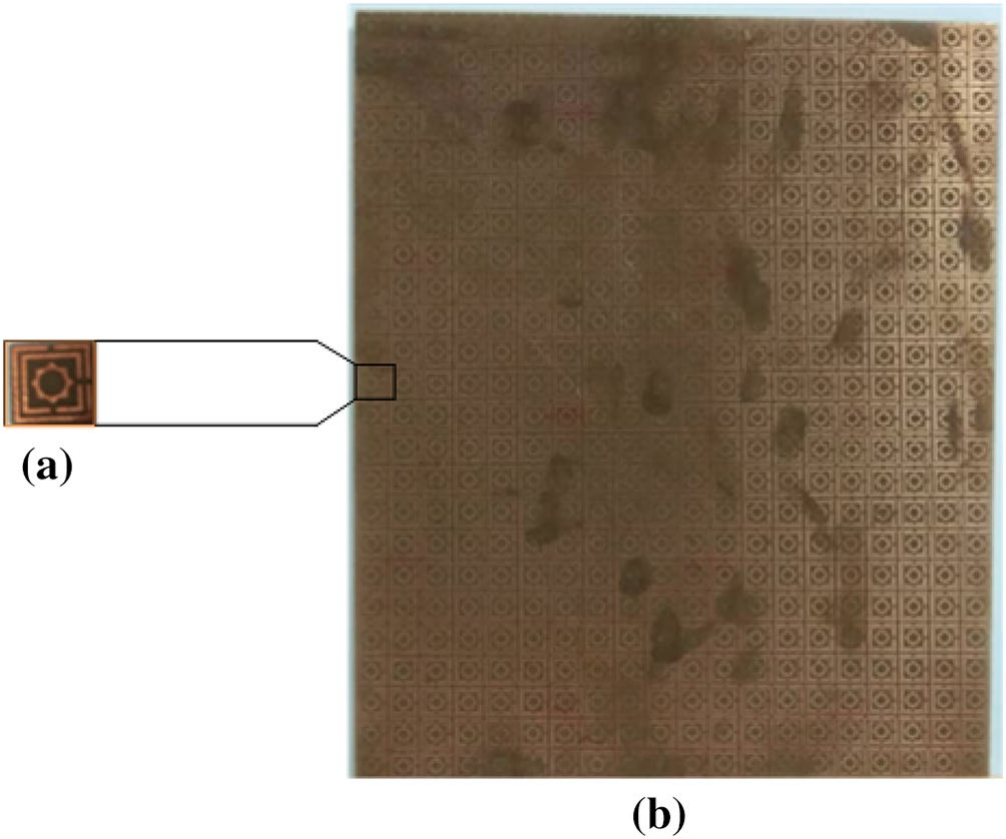
Fabricated prototype of MAA (**a**) unit cell (**b**) array.

**Figure 22:**
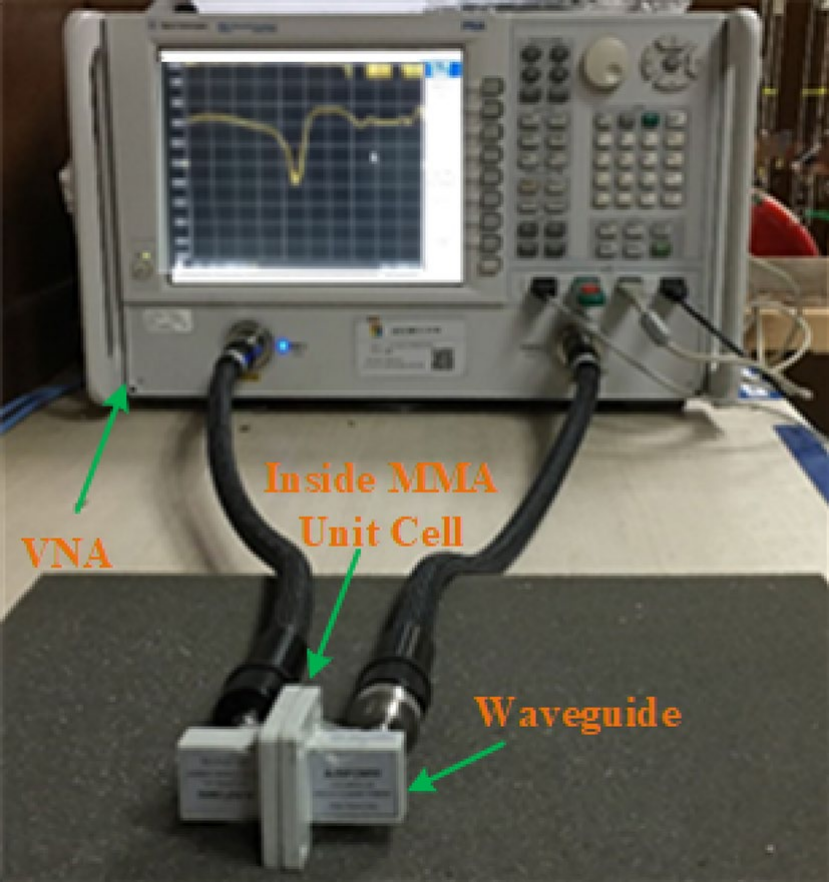
MAA measurement procedure of the unit cell.

**Figure 23:**
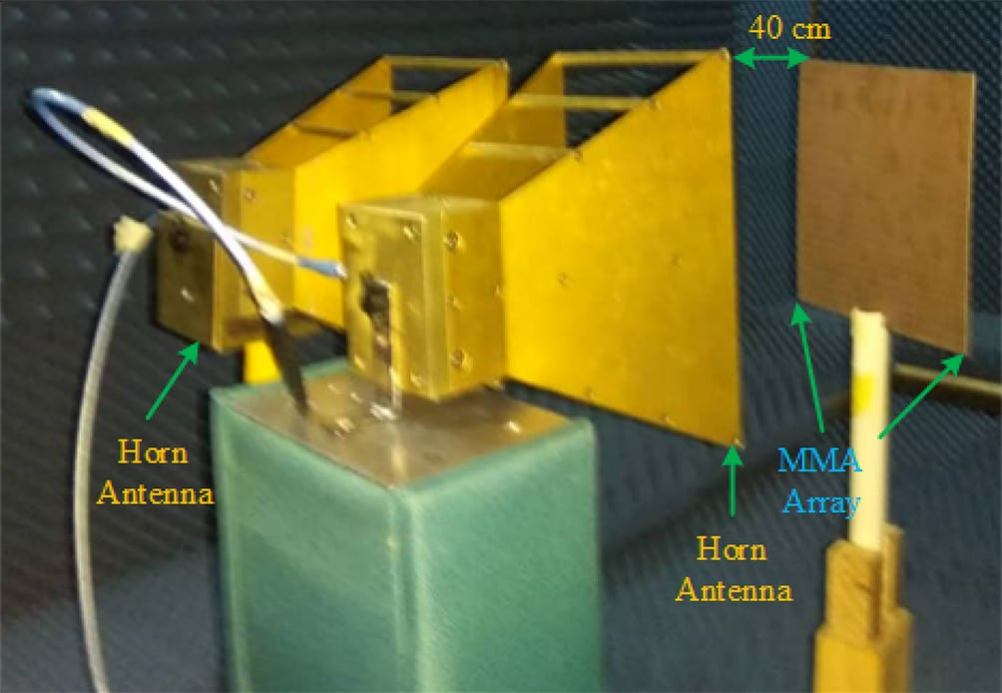
Array measurement procedure of the MAA.

First, we confirmed the proposed MAA unit cell and array design. Then we have produced the arrangement by using the LPKF Laser and Electronics AG, Promat E33 model Computerized Numerical Control (CNC), and printed circuit board (PCB) machine. Produced fabricated unit cell and array structure have been presented. A unit cell and 24 × 20 (192 × 160 mm^2^) array fabricated prototype is revealed in Fig. 21a,b. Since 3.80 GHz is the first resonance frequency of this MAA unit cell's absorption peak, the absorbed signal wavelength is 78.95 mm this frequency. The value of the wavelength of MAA will continue to decrease as the frequency increases. As a result, the array's designated prototype makes sure its greater length contrasted to the incident signify wavelength. Owing to this pattern's more significant imprint, there is a strong probability of better response during the measurement. Several pairs of waveguides have been applied to determine the unit cell result for different frequency ranges. Figure 22 shows the unit cell measurement setup, where the MAA cell has been positioned between one pair of waveguides. Using the waveguide and vector network analyzer first measured the real and imaginary value of S_11_ and S_21,_ and using these values in the MATLAB Code, we have got the absorption result. The measured absorption graph of the designed MAA is depicted in Fig. 23, showing the peaks of absorption 96.97%, 92.34%, 91.43%, 99.03%, and 98.79% at the frequencies of 3.84, 5.73, 8.48, 10.62, and 16.01 GHz, respectively. The simulated and measured results of the MAA unit cell look very similar. A slight discrepancy is seen between the simulated and measured results for the frequency of resonance and magnitude value of the MAA. To measure the MAA unit cell, we have used several pairs of waveguide model. These waveguides model and frequency range are given in Table 10.

**Table 10:** Various pairs of waveguide model and range of frequencies.

Port model of waveguides	Measuring frequency range (GHz)
229WCA3.5	3.30–4.90
159WCAN	4.90–7.05
112WCAS	7.05–10.0
75WCAS	10.0–15.0
51WCAS	15.0–22.0

**Figure 24:**
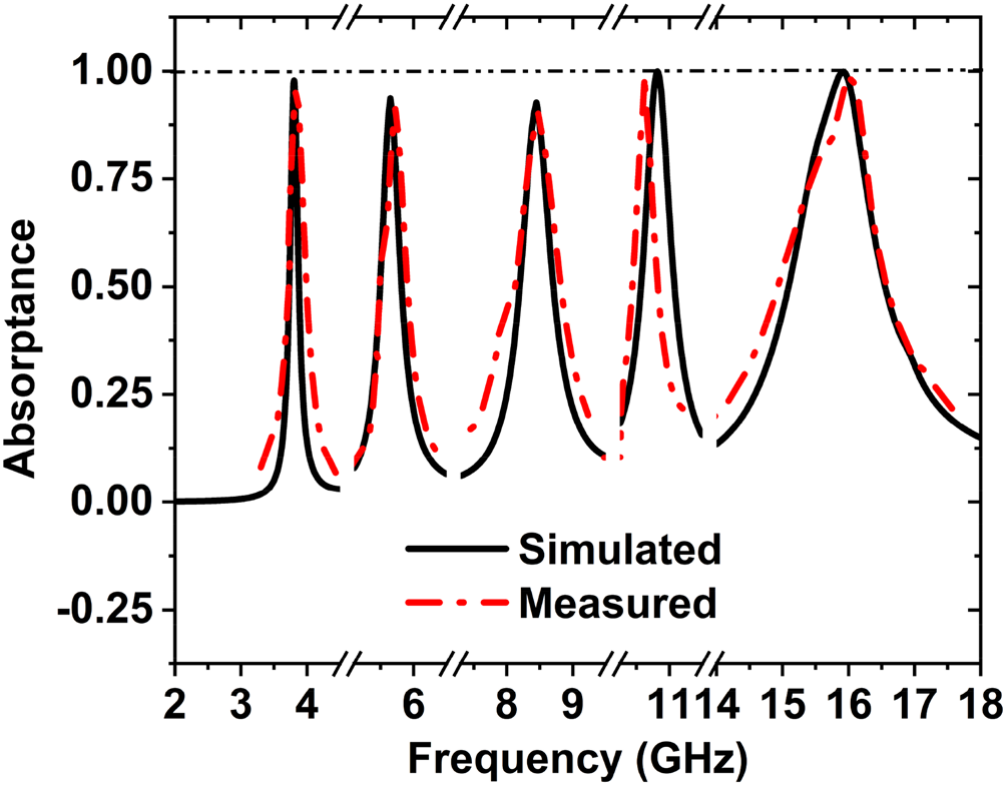
Simulated and measured absorption graph of the MAA nit cell.

The array prototype has been kept on one side of the horn antennas for measurement purposes, as shown in Fig. 24. The operating lower frequency is 700 MHz, and the higher frequency is 18 GHz of these horn antennas and formed in the shape of double edges guided. The whole volume of the antenna is 13.9 × 24.4 cm^2^. To measure the array prototype, the distance between the antenna and the prototype will be far field. The far-field distance ≥ 2D2/λ, for an antenna, where D is the aperture antenna dimension, and λ is the wavelength at the lowest operational frequency. The manufactured array prototype is located 40 cm from two horn antennas at a far-field distance; antennas are used simultaneously for transmitting and receiving signal purposes. The array measurement has been taken in the anechoic chamber to avoid the surrounding noise.

**Figure 25:**
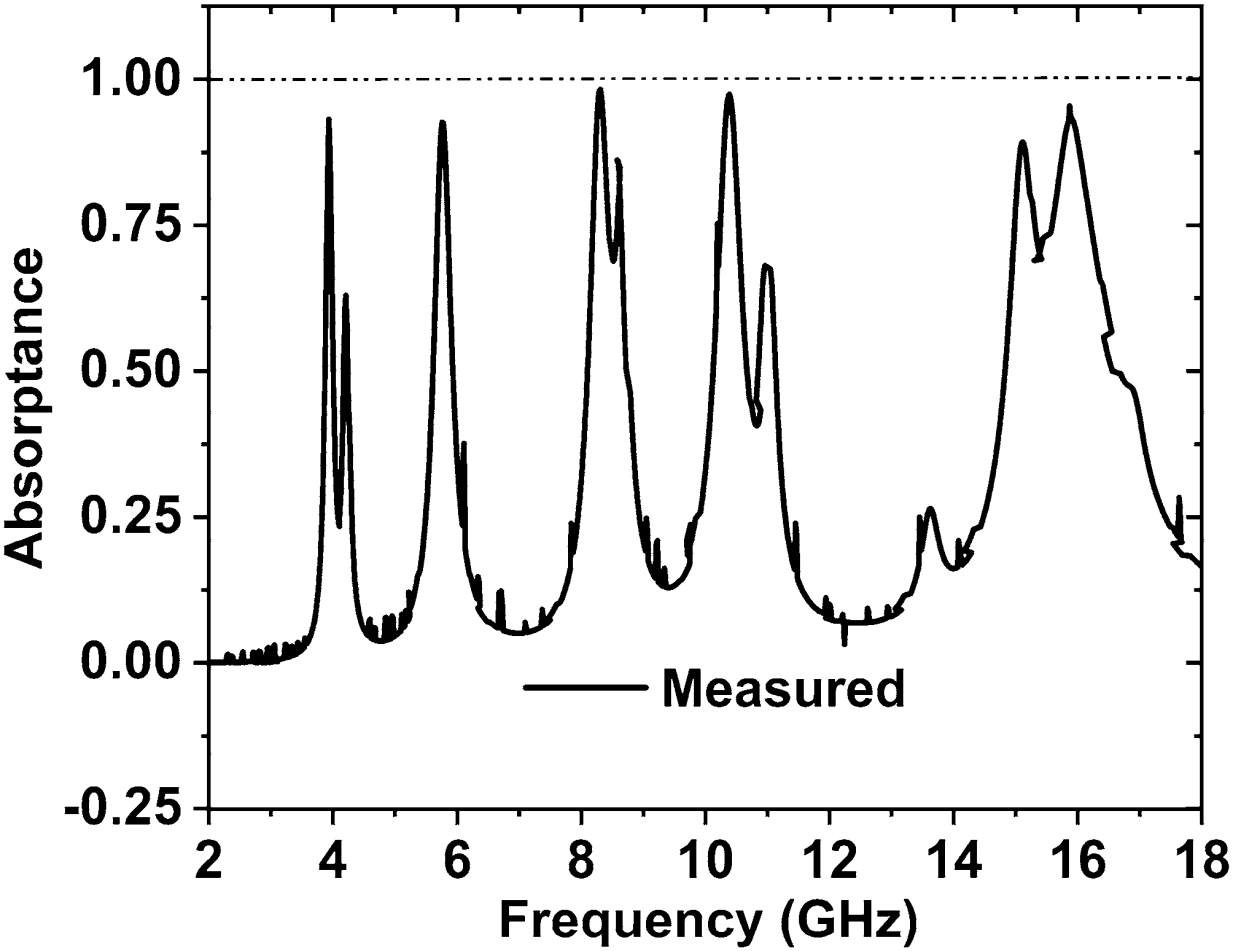
Measured array graph of the suggested MAA.

First, to measure the reflection coefficient, a copper sheet is placed with the same size as the fabricated array size. Subsequently, the blank copper plate is substituted by the fabricated array and measured the response of reflection; the authentic response of S_11_ is achieved by normalization the array data with copper. The measured absorption result of the MAA array is shown in Fig. 25. The array results provide seven absorption peaks of 93.11%, 92.60%, 98.30%, 86.18%, 97.44%, 89.27%, and 95.49% at the resonance frequencies 3.94, 5.76, 8.31, 8.58, 10.39, 15.11, and 15.87 GHz, respectively. The resonance frequencies of the array are slightly shifted compared with the unit cell, and some harmonics are also shown in the array result due to the effect of coupling and some errors in the measurement procedure. Another cause may be frequency shifting, noise, and harmonics for the used lossy cable to connect between horn antennas and VNA.

## EMR calculation

The EMR of a meta-atom expresses the structure's compactness, and it is a vital property of meta-atom. To fulfill the criteria of meta-atom, the value of EMR must be ≥ 4. The calculated formula of EMR can be revealed by the expression $EMR = {{\lambda_{0} } \mathord{\left/ {\vphantom {{\lambda_{0} } L}} \right. \kern-\nulldelimiterspace} L}$, here $\lambda_{0}$ and $L$ are the wavelength and MAA unit cell large dimension, respectively^47^. Since 3.80 GHz is the first peak of absorption, so the value of EMR is 9.87 of the recommended MAA unit cell. A comparison among the proposed MAA unit cell and some other existing MMA papers are given in Table 9. This comparison's main parameters are unit cell size, absorption points, frequencies where highest absorption occurs, EMR, cover bands. From reference ^11,14,21,22,26,31^ we can realize that the dimension of this MMA unit cell is high, the number of resonance frequency, EMR, and cover bands are low, so our proposed MAA unit cell performance is better than the reference^11,14,21,22,26,31^. Though the MMA unit cell dimension of reference^27,29^ are small compared to the proposed MAA unit cell, the number of resonances, EMR, and frequency bands are low than the designed cell, so the suggested MAA unit cell performance is good quality than reference^27,29^. Since the size of the reference ^13,20^ are equal to our fabricated MAA unit cell, but the other parameters, such as EMR, are low, and the covering band is low. The number of resonance frequencies is low; hence, the MAA’s performance is best rather than the reference ^13,20^. Hence the recommended MAA shows a better performance than some published works mentioned in Table 11.

**Table 11:** Comparison of performance among proposed MAA unit cell and some other published MMA unit cell.

References	Year	Size physical size (mm) electrical size ($\lambda_{0}$)	Resonance frequencies (GHz)	EMR	Percentage of peak absorptance	Covering band
^29^	2017	8 × 6.5 0.12λ0 × 0.09λ0	4.20, 9.35, 11.47	8.94	99.68, 99.47, 99.41	C-, X-
^22^	2018	19 × 19 0.88λ0 × 0.88λ0	13.77, 15.30	1.13	99.60, 99.15	X-, Ku-
^13^	2019	8 × 8 0.42λ0 × 0.42λ0	15.53, 27.25	2.41	98.37, 80.08	Ku-, K-
^21^	2017	10 × 10 0.14λ0 × 0.14λ0	3.67, 8.58, 10.16, 14.92	8.14	96.16, 99.16, 99.74, 98.76	S-, X-, Ku-
^14^	2016	20 × 20 0.28λ0 × 0.28λ0	4.35, 6.67, 8.57, 10.65	3.45	98.50, 97.7, 94.80, 96.01	C-, X-
^11^	2017	35 × 35 0.74λ0 × 0.74λ0	6.45,7.67	1.33	~ 95.10	S-
^31^	2019	15.6 × 15.6 0.22$\lambda_{0}$ × 0.22$\lambda_{0}$	4.2, 5.6, 7.7	4.58	99.6, 99.9, 99.7	C-
^20^	2020	8** × **8 0.11$\lambda_{0}$ × 0.11$\lambda_{0}$	4.1, 6.86, 11.3, 13.45	9.15	97.5, 9.1, 99.5, 99.95	C-, X-, Ku-
^27^	2019	6 × 6 0.35λ0 × 0.35λ0	18.25, 21.20, 24.61, 26.16	2.75	94.44, 92.93, 83.71, 93.56	Ku-, K-
^26^	2020	10 × 10 0.38 × 0.38$\lambda_{0}$	11.30, 14.12, 14.22, 17.80	2.64	94.64, 95.59, 97.10, 75.57	X-, Ku-
Proposed	2021	8 × 8 0.101$\lambda_{0}$ × 0.101$\lambda_{0}$	3.80, 5.65, 8.45, 10.82, 15.92	9.87	97.87, 93.65, 92.67, 99.95, 99. 86	S- , C-, X-, and Ku-

## Conclusion

In this article, an SNG meta-atom absorber has been constructed by two square SRR enclosed one-star ring resonator for the penta band microwave applications. The suggested MAA indicates five peaks of absorption of 97.87%, 93.65%, 92.66%, 99.95%, and 99.86% at 3.80, 5.65, 8.45, 10.82, and 15.92 GHz resonance frequency which covers S-, C-, X-, and Ku- band. FR-4 low-cost material has been used as a substrate to make the MAA unit cell which physical dimension is 8 × 8 mm^2^. The unit cell shows SNG performance along with five resonances of S_11_ at 3.80, 5.65, 8.45, 86, 10.82, and 15.92 GHz. In addition, the Q-factors are calculated to determine the selectivity within the desired bands at each resonance frequency. The ADS software has been used to verify with the simulated S_11_ result obtained from the CST-2019 software of the equivalent circuit. The measured result of the fabricated prototype has been a good match with the simulated result. The output of different array configuration of 1 × 2, 2 × 2, and 4 × 4 was taken by the simulation and 24 × 20 array arrangement has been measured and presented. The value of EMR is 9.87, and quality factor (Q factor) of the designed MAA unit cell is 30.41. Since the unit cell provides high EMR, excellent quality factor maximum absorption and the cell size is compact so the recommended MAA can be effectively used as a multiband microwave application.
